# Progress in Surface Modification of SnO_2_ Electron Transport Layers for Stable Perovskite Solar Cells

**DOI:** 10.1002/smsc.202200108

**Published:** 2023-04-12

**Authors:** Jue Gong, Yupeng Cui, Faming Li, Mingzhen Liu

**Affiliations:** ^1^ School of Materials and Energy University of Electronic Science and Technology of China Chengdu 611731 P. R. China

**Keywords:** defect passivation, operational stabilities, perovskite solar cells, SnO_2_ electron transport layers, surface modifications

## Abstract

The photovoltaic (PV) performance of perovskite solar cells (PSCs) has rapidly advanced in the recent years; yet, the stability issue remains one of the last‐mile challenges on the road to commercialization. Charge transport layers and their interfaces with perovskites stand for critical tuning knobs that determine the device stability of PSCs. This review focuses on the effects of modification of SnO_2_ electron transport layers (ETLs) on the interfacial physicochemical properties and stability of PSC devices. In detail, the intrinsic defects, surface hydroxyls, and nonuniform morphology of SnO_2_ will negatively impact its interfacial physicochemical properties, thus degrading the device stability of PSCs. To tackle these existing issues, three modification approaches, such as surface morphology control, surface physicochemical modifications, and surface composite‐structure design, are categorized. Lastly, future perspectives in further promoting the stability of PSCs from SnO_2_ ETLs are raised based on the currently unresolved issues from both material and device levels.

## Graphical abstract

The SnO_2_ electron transport layers/perovskite interface affects the device stability of perovskite solar cells through its defect and interfacial energy profiles. Tailored surface modification approaches have therefore been developed to suppress the surface defects and energy‐level alignments at the SnO_2_/perovskite interfaces through morphological control, physicochemical modification, and composite‐structure designs.

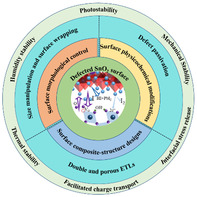

## 1 Introduction

Since the first invention of perovskite solar cells (PSCs), its power conversion efficiency (PCE) has rapidly surged to 25.8%,^[^ ^1^ ^]^ with flexible PSCs also reaching a high PCE of 23.6%.^[^ ^2^ ^]^ However, stability of PSCs is still considerably inferior that have rendered them not able to fulfill the commercialization standards at the moment.^[^ ^3^ ^]^ So far, the approaches adopted to mitigate the instability issues of PSCs include enhancements of the intrinsic stability of perovskite light‐harvesting materials, modification of functional layers, tailored interfacial engineering, and device encapsulations.^[^ ^4^ ^]^ Due to high ion mobility and intrinsic surface defects (e.g., dangling bonds, uncoordinated/undercoordinated sites) of perovskite materials,^[^ ^5^ ^]^ the neighboring charge transport layers that sandwich perovskite in between serve the critical roles of not only regulating but also preserving the physical/chemical integrity of perovskites. Out of the charge transport layers, hole transport layers (HTLs) such as spiro‐OMeTAD and PTAA with tailored molecular functions and interfacial properties^[^ ^6^ ^]^ have contributed to improved stabilities of PSCs under moisture and thermal stresses. Yet, the complementary aspects such as optical, chemical, and mechanical stabilities, as mediated by electron transport layers (ETLs), remain relatively underexplored in PSCs with n–i–p architecture.

**Figure 1:**
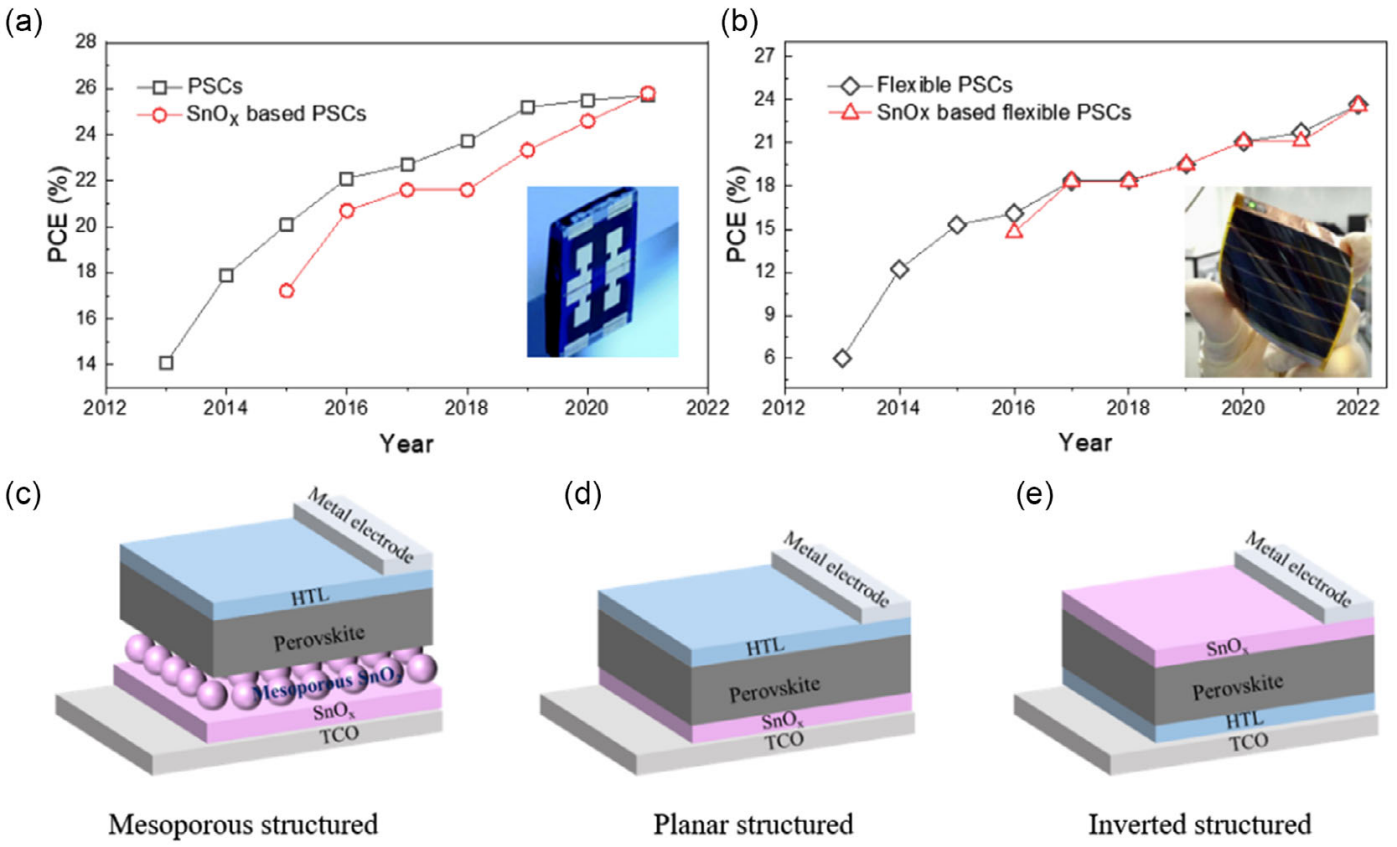
Research progress of PSCs based on different substrates, ETL and device structures. a) Evolution of top‐performance PSCs on rigid substrates.^[^ ^1^, ^5^, ^14^ ^]^ and b) on flexible substrates.^[^ ^2^, ^63^ ^]^ Performance of devices based on SnO_2_ ETLs is shown by red curves. Inset: photographs of typical rigid and flexible PSCs.[^14^, ^64^] c–e) PSCs employing mesoporous (c), planar (d), and inverted (e) device structures.

Among the many ETLs, inorganic ETLs such as SnO_2_, TiO_2_, Zn_2_SnO_4_ have desirable properties such as high optical transparency, structural, and thermodynamic stabilities. Due to these advantages, ETL/perovskite/HTL multilayer stacks can function to effectively protect perovskite materials, constituting pivotal pathways to improve stabilities at device level in response to external stress conditions. Representing an ETL that has retarded chemical reactivity or photocatalytic activity with perovskite materials, SnO_2_ is a promising candidate for operationally stable and high‐performance PSCs. Moreover, it possesses much greater electron mobility for inhibiting interfacial carrier accumulation and the consequent current–voltage (*J*–*V*) hysteresis in solar cells; the moisture‐resistant, UV‐tolerant natures of SnO_2_ even make it potentially viable for long‐term, steady‐state power output of the incorporated PSCs.^[^ ^7^ ^]^ So far, PSCs with rigid and flexible substrates have achieved top solar‐to‐electricity performance with SnO_2_ being the ETLs (**Figure** **1**). Nonetheless, the self‐doped defects at the surface of SnO_2_ (such as Sn and O vacancies, surface hydroxyls, etc.) exert profound impact to the performance and stability of PSCs.^[^ ^8^ ^]^ In detail, hydroxyl groups (–OH) and dangling bonds on SnO_2_ surface decrease the formation energy of oxygen vacancies and promote oxygen diffusion toward perovskite lattice,^[8a,b,d]^ while causing proton movements from the organic cations, thereby degrading the thermal stabilities of perovskite materials.^[^ ^9^ ^]^As such, it can be seen that the major degradation mechanism of SnO_2_‐based PSCs of both the chemical properties and PV performance is mainly attributed to the defective buried interfaces between SnO_2_ ETLs and perovskites, thus making the modifications at the SnO_2_ surface/interfacial locations extremely crucial for stability enhancement in PSCs. To date, most article reviews of SnO_2_‐based PSCs mainly focus on the influence of bulk doping and surface modification of ETLs in the PV performance,^[^ ^10^ ^]^ with inner connections between the surface/interfacial modifications and long‐term stabilities under moisture, elevated temperatures, and light irradiation conditions lacking in‐depth analysis. The consolidation of this scientific building block, therefore, will inspire relevant ideas to further improvements of stability at both material and device levels.

This review focuses on surface morphology, defects, interfacial stress and energetics of SnO_2_, and the corresponding effects in device stability of PSCs. Based on the underlying structure–property relationships, we further generalize and categorize three surface modification approaches—morphology control, physicochemical modifications, and composite‐structure design. We first introduce the optical and electronic properties of SnO_2_ ETLs in Section 2, based on which we proceed to the modification approaches of SnO_2_/perovskite interfaces of PSCs in Section 3. This work will advance the understanding of the important and affecting roles of SnO_2_/perovskite interfaces in stabilizing PSCs under long‐term PV operation.

## 2 Physical Properties of SnO_2_ Electron Transport Layers

Since ETLs serve the roles of electron extraction and transport in PSCs, it is desirable for them to exhibit high charge extraction potential, less charge accumulation, and less charge recombination when forming interfaces with perovskite films. In addition, ETLs are desired to possess minimum parasitic absorption so to maximize the light absorbable by perovskite active layer. In this section, we will review the properties of SnO_2_ from the four aspects—photoelectric properties, energy band structures, film formation, and defect properties.

### 2.1 Optoelectronic Properties

#### 2.1.1 Large Bandgap and Optical Transmittance

**Figure 2:**
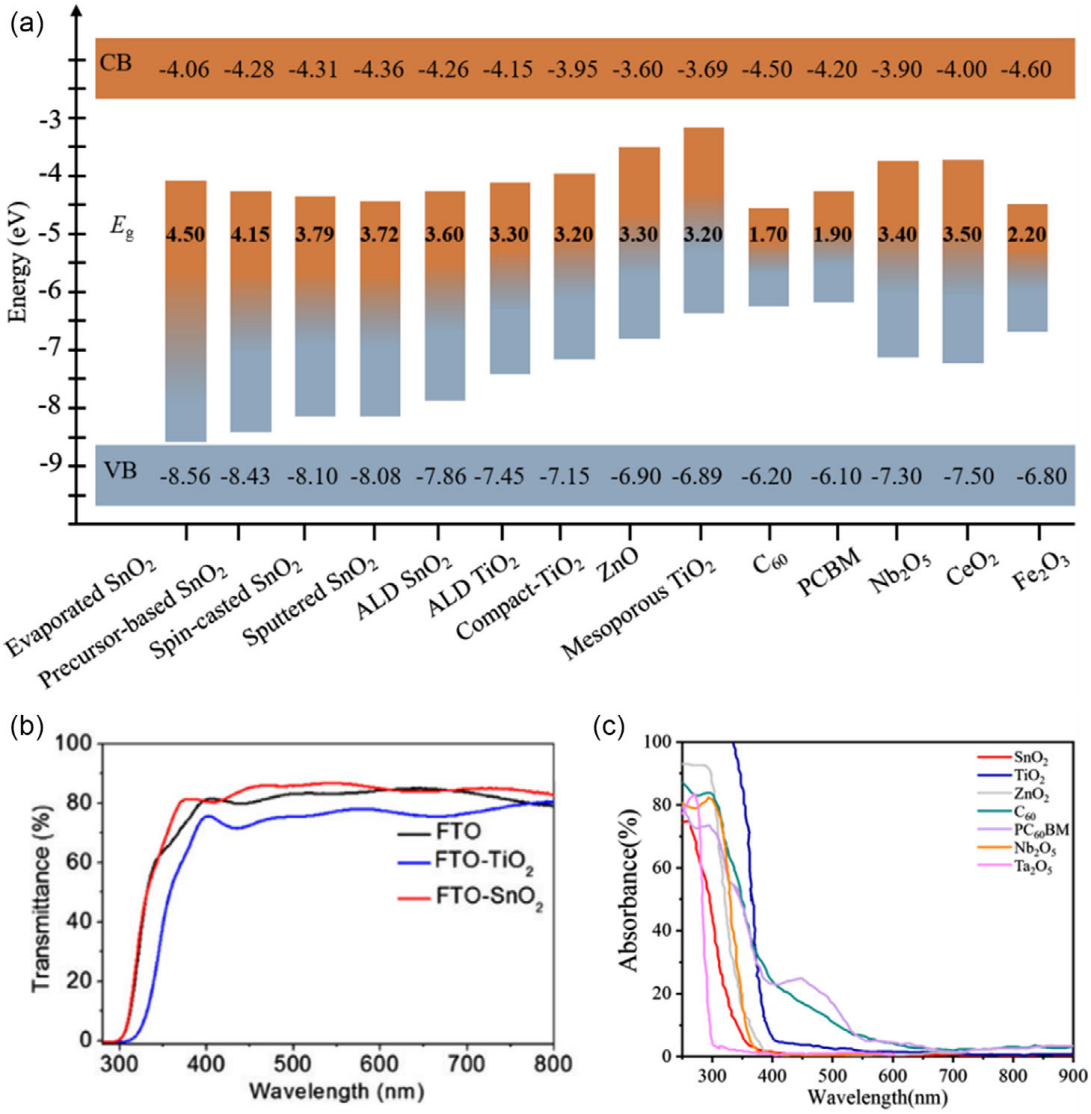
Comparison of optical properties of different ETLs. a) Energy band diagrams of the most commonly applied ETLs including SnO_2_,^[^ ^20^ ^]^ TiO_2_,[^20^, ^65^] ZnO,^[^ ^66^ ^]^ C_60_,^[^ ^67^ ^]^ PCBM,^[^ ^68^ ^]^ Nb_2_O_5_,^[^ ^69^ ^]^ CeO_2,_^[^ ^70^ ^]^ and Fe_2_O_3_.^[^ ^71^ ^]^ b) Optical transmittance spectra of FTO (black), FTO‐TiO_2_ (blue), and FTO‐SnO_2_ (red) films.^[14a]^ b) Reproduced with permission.^[14a]^ Copyright 2015, American Chemical Society. c) Optical absorbance spectra of various ETLs.[^10^, ^72^]

The optical bandgap of a semiconductor directly dictates the cutoff energy of photons that can be absorbed. SnO_2_ has bandgaps ranging from 3.60 to 4.50 eV according to different fabrication methods (**Figure** **2**a),^[10b]^ which renders it transparent to most visible region when compared with other ETL materials, such as TiO_2_, ZnO, and PCBM (Figure 2b,c). In detail, it is the defects, crystallinity, and/or particle sizes from different synthetic approaches that give rise to the different optical bandgaps of SnO_2_, where the types and amounts of defects directly determine the relative coordination between Sn and O elements, thereby affecting the electron orbital overlap and thus the energy gap,^[^ ^11^ ^]^ as similar to oxygen‐deficient TiO_*x*_ materials.^[^ ^12^ ^]^ Characteristically, SnO_2_ has high transmittance (*T*) of solar spectrum above 80% within the spectral range of 400–800 nm (Figure 2b), and thus constitutes minimum parasitic absorption and ensures a full exploitation of solar irradiation by perovskite light‐harvesting layers, where the relationship between *T* and absorption coefficient (*α*) is described as^[^ ^13^ ^]^

(1) $$T\approx e^{-\alpha t}$$

where *t* stands for film thickness, where SnO_2_ film typically will not exceed 20–30 nm. Along with the very small *α* at wavelengths where light is transmissible, the transmittance by SnO_2_ is typically closed to unity.

The refractive index is also an important factor affecting the light transmittance. Since SnO_2_ ETLs have particularly low refractive index in the visible region, and the overall refractive index in combination with TCOs (FTO, ITO) is also much smaller than other types of ETLs.^[^ ^14^, ^15^ ^]^ As such, sunlight can pass through SnO_2_ ETL substrates without much loss solely due to the prolonged optical loss by refraction.

#### 2.1.2 Electrical Properties

**Figure 3:**
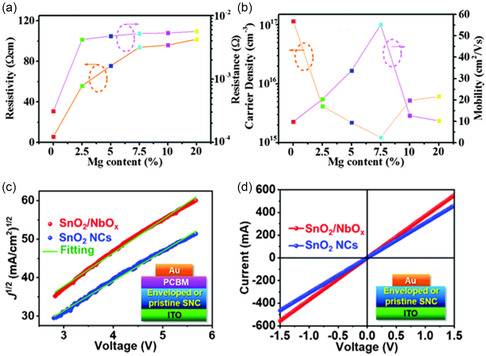
Electrical properties of SnO_2_ ETLs with different elemental doping and functionalization. a) Electrical resistivity/resistance and b) carrier density/mobility at different Mg doping contents.^[^ ^16^ ^]^ a,b) Reproduced with permission.^[^ ^16^ ^]^ Copyright 2016, Royal Society of Chemistry. c) Mott–Gurney plots and d) *I*–*V* plots of NbO_*x*_‐enveloped SnO_2_ and pristine SnO_2_ NCs as ETLs.^[^ ^17^ ^]^ c,d) Reproduced with permission.^[^ ^17^ ^]^ Copyright 2021, Royal Society of Chemistry.

The intrinsic electrical properties of SnO_2_ ETLs govern the electron transport process herein and the eventual charge collection at the conductive electrodes, as evaluated by electrical resistivity, electron density, and mobility. Pristine SnO_2_ exhibits relatively low resistivity/resistance. However, obvious rise of electrical resistivity/resistance and reduction of carrier density are resulted upon Mg metal ion doping, which reach above 100 Ω·cm resistivity and 10^15^ to 10^16^ cm^−3^ carrier concentrations in the range of 2.5%–20% Mg doping, respectively, despite considerable improvement of electron mobility that falls within 10–55 cm^2^ V^−1^ s^−1^ (**Figure** **3**a,b).^[^ ^16^ ^]^ By engineering SnO_2_ ETLs for simultaneously improved mobility and conductivity, it was previously demonstrated that SnO_2_ nanocrystals (NCs) exhibited boosted charge extraction after functionalized with NbO_*x*_ wrapping shells, leading to synergistically enhanced electron mobility and conductivity with 900–3000 mA cm^−2^ current density within 3–5 V electrical bias (Figure 3c,d).^[^ ^17^ ^]^ The increased mobility will reduce charge accumulation at the ETL/perovskite interface for remarkably suppressed hysteretic behaviors of solar cell devices. The exceptional electron mobility and conductivity after interfacial treatments thus indicate the promising electrical performance of SnO_2_ being ETLs applicable in PSCs.

### 2.2 Energy‐Level Alignment with Perovskites

**Figure 4:**
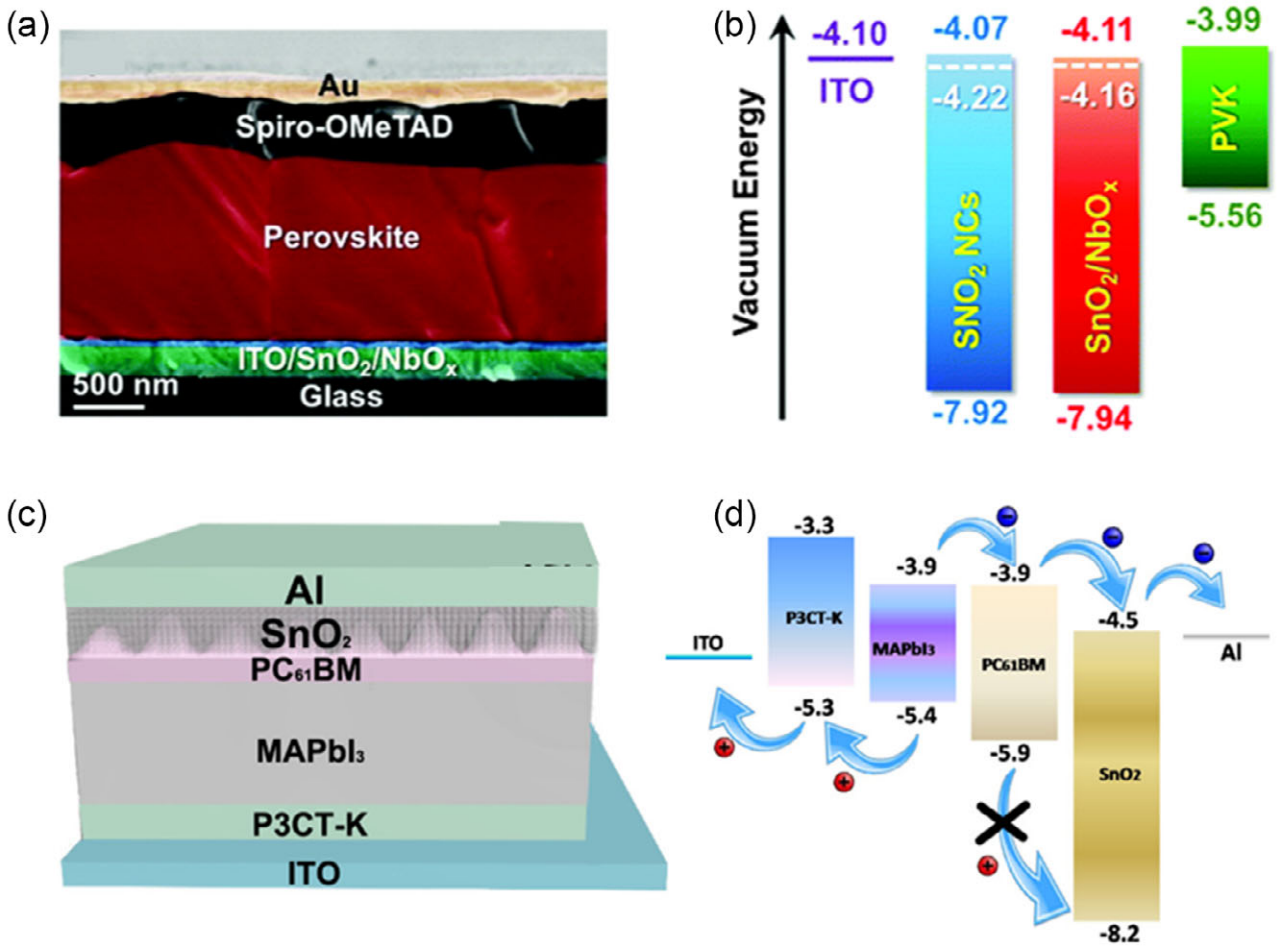
Device structure dependent energy‐level alignments in PSCs with SnO_2_ ETLs. a) Cross‐section illustration of a regular n–i–p PSC and b) corresponding energy band diagrams of all functional layers.^[^ ^17^ ^]^ a,b) Reproduced with permission.^[^ ^17^ ^]^ Copyright 2021, Royal Society of Chemistry. c) Schematic depiction of inverted p–i–n PSC and d) corresponding energy band diagrams of the functional layers.^[^ ^18^ ^]^ c,d) Reproduced with permission.^[^ ^18^ ^]^ Copyright 2018, American Chemical Society.

Conduction bands (CBs) of SnO_2_ are notably lower in energy levels with respect to the perovskites, which renders this material a good electron extractor while effectively inhibiting hole injection from perovskite layer, thus preventing the undesirable carrier recombination. Most importantly, the CB offsets between SnO_2_ and perovskites are small as compared to other ETL materials, which can therefore avoid large overpotential and *V*_oc_ loss while ensuring a facilitated charge transfer, as shown by the schematic band diagrams of a typical n–i–p device (**Figure** **4**a,b).^[^ ^17^ ^]^ The ideal properties of SnO_2_ are further exemplified by the fact that its energy band positions are highly tunable upon specific surface modification (e.g., NbO_*x*_ surface wrapping) as shown in Figure 4b. This regulative approach is highly useful as perovskites with different compositions are characteristic with Fermi levels, CBs, and valence bands (VBs) at different energy levels; adjusting the energy band positions of SnO_2_ thus become very necessary in realizing cascaded energetics for carrier extraction from perovskite absorbers. Moreover, the realization of SnO_2_ as ETLs is not only limited to regular n–i–p device structure, where SnO_2_ ETLs can be fabricated on top of perovskites in p–i–n inverted devices through spin‐casting relatively nonpolar solutions containing SnO_2_ NCs (Figure 4c).^[^ ^18^ ^]^ Such device architecture, when composited with PCBM interlayer, can further achieve cascaded energy band alignments for minimized charge potential loss (Figure 4d). As device hysteresis is mainly influenced by the two interfaces between perovskite and charge transport layers in ETL/perovskite/HTL stacks, it is further believed that the matched energy‐level alignment between SnO_2_ and perovskite will also contribute to the mitigation of potential hysteretic behaviors of PSC devices.

### 2.3 Film Formation and Low‐Temperature Processing of SnO_2_ Electron Transport Layers

For the preparation of SnO_2_ ETLs, various synthetic methods can be adopted such as chemical bath deposition (CBD),^[^ ^19^ ^]^ atomic layer deposition (ALD),[^20^, ^21^] solution spin‐coating, dip coating, slot casting, spray coating, and so on.^[^ ^22^ ^]^ Different synthesis approaches can result in SnO_2_ films with different morphological properties that affect the stability of perovskite films and PSCs,^[^ ^23^ ^]^ where a summary of device stability regarding PSCs with SnO_2_ ETLs with different synthesis approach is explicated in **Table** **1**.

**Table 1:** Progress in the stability of perovskite solar cells based on SnO_2_ ETLs with different synthetic approaches

[Year]	ETL processing	Device structure	Stability	PCE1a)	Ref.
				PCE2b)	
2015	Slot‐die coat SnO_2_	ITO/SnO_2_/PVSKc)/Spiro‐OMeTAD/Ag	Unencapsulated, AM1.5G, ambient air, 30 days	13.0%	[73]
				≈11%	
2016	SCd)‐CBD SnO_2_	FTO/SnO_2_/PVSK/Spiro‐OMeTAD/Au	Unencapsulated, dry air, 90 days	20.70%	[14b]
				>20%	
2017	Hydrothermal SnO_2_	FTO/SnO_2_/PVSK/Spiro‐OMeTAD/Au	Unencapsulated, air, RT, 500 h	18.31%	[74]
				16.47%	
2018	SC SnO_2_	ITO/SnO_2_/PVSK/Spiro‐OMeTAD/Au	Unencapsulated, ambient atmosphere, 2880 h	21.6%	[14d]
				19.87%	
2018	CBDe) SnO_2_	FTO/SnO_2_/ZWf)/PVSK/Spiro‐OMeTAD/Au	Unencapsulated, 85 °C, RH = 85%, 140 h	21.43%	[20b]
				19.93%	
2018	SC SnO_2_	FTO/SnO_2_/KCl/PVSK/Spiro‐OMeTAD/Au	Unencapsulated, N_2_, 25 °C, RH = 50%–70%, 720 h	20.5%	[75]
				18.45%	
2019	SC SnO_2_	FTO/Ru + SnO_2_/PVSK/Spiro‐OMeTAD/Au	25 °C, 2000 h	22%	[76]
				21.34%	
2019	SC SnO_2_	ITO/SnO_2_/PS^g)^/ PVSK/PS/Spiro‐OMeTAD/Au	Inner‐encapsulated, 5 days	21.89%	[53]
				21.23%	
2019	SC SnO_2_	ITO/SnO_2_/PVSK/Spiro‐OMeTAD/Au	Unencapsulated, outdoor irradiation, 180 h/UV irradiation, 500 h	21.01%	[77]
				19.75%/16.8%	
2019	SC SnO_2_	ITO/SnO_2_/PVSK/MoO_3_/Spiro‐OMeTAD/Au	Unencapsulated, RH = 40%‐60%, 25 °C, dark, 1000 h	22.77%	[7b]
				21.63%	
2020	SC SnO_2_	ITO/In_2_O_3_/SnO_2_/PVSK/Spiro‐OMeTAD/Au	Unencapsulated, N_2_, 25 °C, 80 days	23.24%	[55]
				22.66%	
2020	CBD SnO_2_	FTO/SnO_2_/PVSK/Spiro‐OMeTAD/Au	Unencapsulated, 85 °C, RH = 85%, 1056 h/full‐sun illumination, 1620 h	24.59%	[14e]
				23.11%/24.09%	
2021	CBD SnO_2_	FTO/SnO_2_/FAPbI_3_/Spiro‐OMeTAD/Au	Continuous light exposure, 500 h	25.8%	[1]
				23.22%	
2021	SC SnO_2_	FTO/SnO_2_/PVSK/Spiro‐OMeTAD/Au	Unencapsulated, 6–25 °C, RH = 20%–30%,air, AM1.5G, 2000 h	22.08%	[78]
				20.76%	
2021	SC SnO_2_	ITO/SnO_2_/PVSK/Spiro‐OMeTAD/Au	50% increase of adhesion toughness Unencapsulated, N_2_, 4000 h	21.4%	[49]
				17.12%	
2021	SC SnO_2_	ITO/SnO_2_/BGCl/PVSK/HTL/Ag	Unencapsulated, 20 °C, RH = 30%, dark, 500 h	24.4%	[42]
				23.18%	
2021	SC SnO_2_	FTO/SnO_2_/PVSK/Spiro‐OMeTAD/Au	Unencapsulated, N_2_, 40 °C, one‐sun, 1000 h	22.2%	[62]
				18.2%	
2021	SC SnO_2_	FTO/TiO_2_/SnO_2_/PVSK/Spiro‐OMeTAD/Au	Unencapsulated, N_2_, RH = 0%, AM1.5 , 1000 h	97% of initial PCE	[79]
2021	CBD SnO_2_	FTO/SnO_2_/PVSK/Spiro‐OMeTAD/Au	Unencapsulated, 100 mW cm^−2^, AM1.5G, 45 °C, 500 h, MPPTh)	25.4%	[19]
				20.4%	
2022	SC SnO_2_	ITO/SnO_2_/FSA/ PVSK/Spiro‐OMeTAD/Au	Unencapsulated, N_2_, AM1.5G, 50‐60 °C,1000 h, MPPT	24.1%	[80]
				18.08%	
2022	SC SnO_2_	ITO/SnO_2_/ADAA/PVSK/Spiro‐OMeTAD/Ag	Unencapsulated, 60 °C, 1000 h	23.18%	[52]
				18.78%	

a) PCE1: initial PCE;

b) PCE2: retained PCE;

c) PVSK: perovskite;

d) SC: spin coating.

e) CBD: chemical bath deposition;

f) ZW: zwitterion;

g) PS: buffer polystyrene/cap polystyrene;

h) MPPT: maximum power point tracking.

Among the various preparation routes, SnO_2_ is most well known for its low‐temperature solution processing, which does not require elevated temperature conditions for annealing to attain the benign electronic properties, whereas high temperatures between 400 and 500 °C are required to reach good crystallinity and structural integrity of TiO_2_ as ETLs. Such intensive thermal conditions, however, have prevented the deployment of TiO_2_ as ETLs in many device applications with heat‐vulnerable substrates, such as flexible PSCs and silicon HJTs. It is exactly because of its low‐temperature processing nature that renders SnO_2_ ETLs energetically moderate and advantageous in flexible PSCs and large‐area PSCs,^[^ ^24^ ^]^ making them beneficial to the low‐cost, upscaled production of PSCs with pronounced application prospects. Specifically, Qi and coworkers previously fabricated large‐area SnO_2_ ETLs via CBD method and the scalable perovskite solar submodules with aperture area >200 cm^2^, demonstrating a PCE > 15%, while the 5 cm × 5 cm mini‐modules using the same ETL processing exhibited a T_80_ operational stability of 1020 h;^[^ ^25^ ^]^ on the other hand, mini‐modules with 22.8 cm^2^ aperture areas based on room‐temperature‐sputtered SnO_2_ ETLs were likewise fabricated, giving >12% device efficiency with T80 operational lifetime of 515 h.^[^ ^26^ ^]^ These results clearly demonstrate the promising potential of SnO_2_ ETLs in the large‐area photovoltaics with scalable sizes.

### 2.4 Defects in SnO_2_ Electron Transport Layers

Although SnO_2_ is featured with benign properties such as chemical stability, optical transparency, and low‐temperature processability as elaborated earlier, surface and bulk defects nonetheless exist in SnO_2_ that affect the structural and electronic properties of the interfaces and PSC devices. In detail, surface defects include oxygen vacancy (V_O_),[^8^, ^27^] hydroxyl groups (–OH),^[8b,d]^ and impurity ions, while the formation and existence of such defects are related to the surface dangling bonds and uncoordinated/undercoordinated atoms due to the small crystallite sizes and low‐crystallinity nature of SnO_2_ from its low‐temperature processing.^[^ ^9^ ^]^ Although numerous bulk defects, namely V_O_, tin vacancies (V_Sn_), tin (Sn_O_)/oxygen (O_Sn_) antisites, oxygen (O_*i*_)/tin (Sn_*i*_) interstitials, also exist in SnO_2_ ETLs, surface defects rather play more significant roles in affecting the interfacial charge extraction from perovskite absorbers and physical quality of perovskite films deposited on top. To suppress surface defects, efforts have been carried out through surface modification methods to target the large number of dangling bonds and hydroxyl terminal defects on the surface, as detailed in later context.

## 3 Approaches to Improve Stability of PSCs Based on Electron Transport Layers

**Figure 5:**
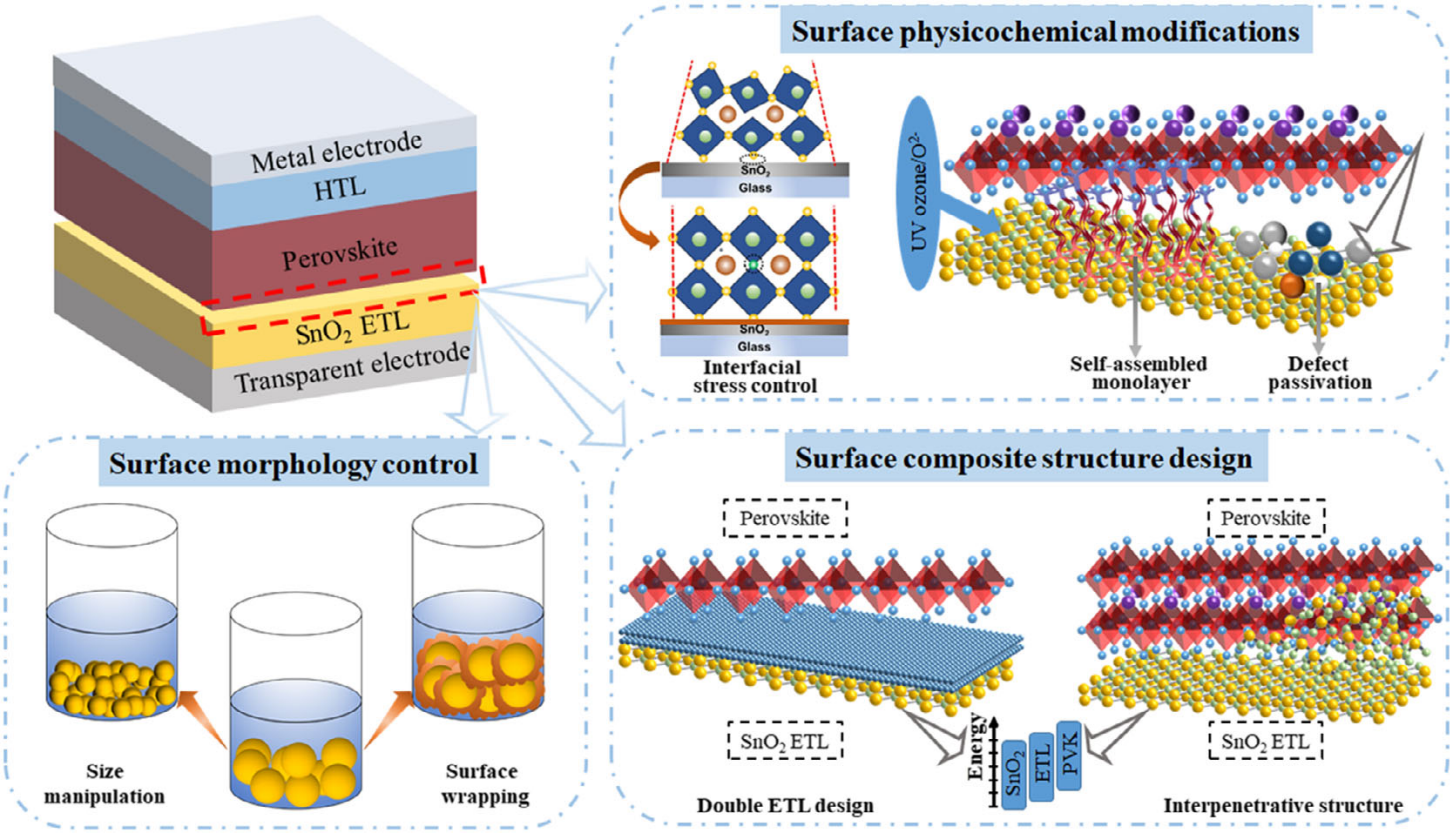
Schematic illustrations of the routes for improving the stability of PSCs by modifying SnO_2_ ETLs. Surface modification strategies are outlined as morphological control, physicochemical modifications, and design of composite‐structure ETLs. Interfacial stress schematic is reproduced with permission.^[^ ^38^ ^]^ Copyright 2021, Elsevier.

Stability is an important performance parameter of PSCs, which is directly related to the commercialization of PSCs.^[^ ^28^ ^]^ Based on the aforementioned analysis, SnO_2_ has been proved to be a suitable ETL in PSCs.^[^ ^29^ ^]^ With various surface defects in SnO_2_ hindering the optimizing of device stability in PSCs,^[^ ^30^ ^]^ we summarize three major pathways to improve the stability of PSCs from the perspective of SnO_2_ ETLs, including surface morphological control, defect passivation, and composite‐structure design, as shown in **Figure** **5**.

### 3.1 Surface Morphological Control

#### 3.1.1 Crystal Size Manipulation in SnO_2_ Electron Transport Layers

**Figure 6:**
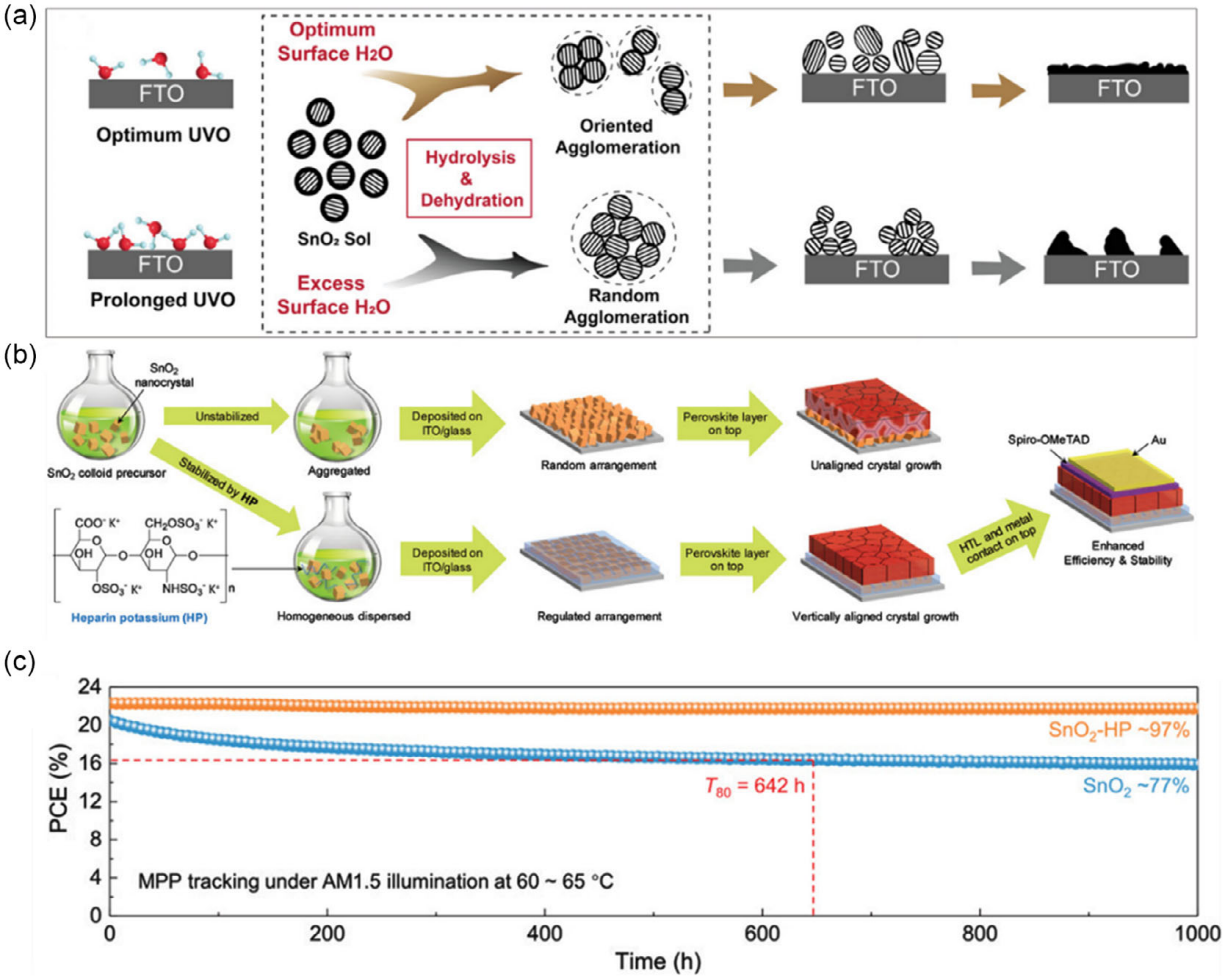
Particle and morphological control of SnO_2_ ETLs. a) Schematic depiction of H_2_O pretreatment through UVO for subsequent deposition of ETLs with different SnO_2_ particle sizes.^[^ ^31^ ^]^ Reproduced with permission.^[^ ^31^ ^]^ Copyright 2019, Wiley‐VCH. b) Processing scheme of polymer‐regulated SnO_2_ NCs for ETLs of high‐performance PSCs.^[^ ^32^ ^]^ c) MPP tracking of solar cell devices based on pristine and polymer‐regulated SnO_2_ ETLs under continuous one‐sun illumination for 1000 h.^[^ ^32^ ^]^ b,c) Reproduced with permission.^[^ ^32^ ^]^ Copyright 2020, Wiley‐VCH.

Targeting the low crystallinity issue of SnO_2_ ETLs as caused by the low‐temperature processing, the in situ regrowth of SnO_2_ NCs was reported via introducing optimum trace amounts of surface adsorbed water on FTO or ITO substrates by UV‐ozone (UVO) treatments (**Figure** **6**a).^[^ ^31^ ^]^ The surface water content exhibited a monotonic increment upon extended UVO treatment duration, but prolonged UVO treatment of TCO substrates led to excessive surface water that caused random agglomeration of SnO_2_ crystallites, thus forming incomplete surface coverage of the underlying FTO substrates required for good PV performance of PSCs (Figure 6a).^[^ ^31^ ^]^ As a result, SnO_2_ ETLs with the optimum morphology and greater crystallinity lead to PSC devices with greater PCE (>20%) with respect to the reference and control device counterparts with less optimized SnO_2_ surface morphology.^[^ ^31^ ^]^ The regulation of SnO_2_ ETLs in affecting device performance and stability of PSCs is also manifested in the uniformity of SnO_2_ nanoparticle sizes. Polymer heparin potassium (HP) was further utilized to realize homogeneous dispersion of SnO_2_ colloidal nanoparticles in solution form, which directly resulted in ordered arrangement of SnO_2_ NCs and perovskite films with vertically oriented grains after depositions (Figure 6b).^[^ ^32^ ^]^ The high‐quality growth of perovskite film on top of HP‐functionalized SnO_2_ ETLs ultimately gave rise to exceptional device stability under continuous one‐sun illumination for 1000 h with negligible performance loss, with devices fabricated on pristine SnO_2_ ETLs showing more than 20% performance degradation relative to HP‐treated counterparts under identical stress conditions (Figure 6c).^[^ ^32^ ^]^

#### 3.1.2 Surface Wrapping of SnO_2_ Electron Transport Layers

**Figure 7:**
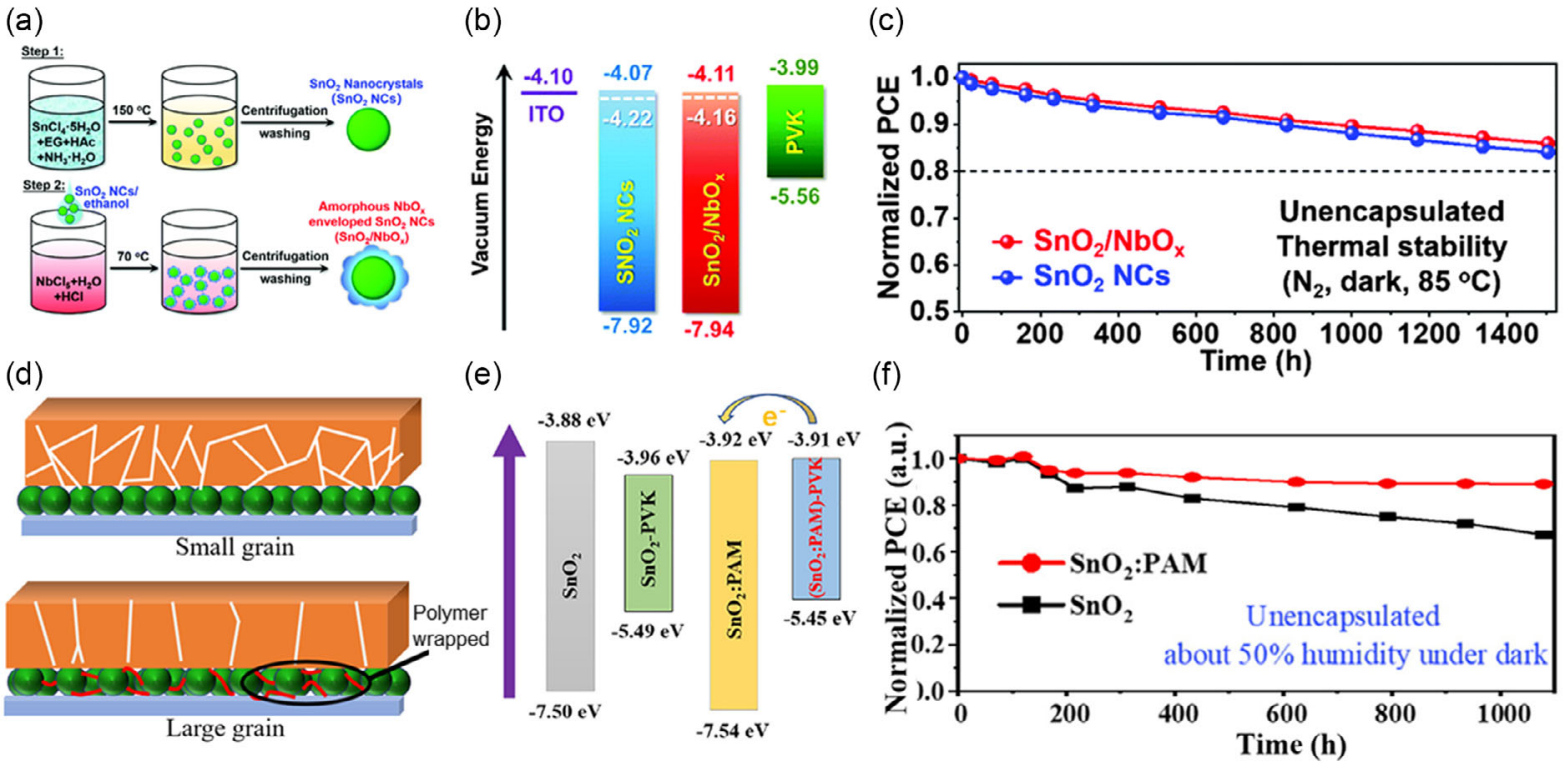
Surface wrapping and functionalizing in SnO_2_ ETLs for stable PSCs. a) Synthesis scheme, b) energy‐level alignment, and c) thermal stability of devices based on pristine and NbO_*x*_‐wrapped SnO_2_ NCs as ETLs.^[^ ^17^ ^]^ a–c) Reproduced with permission.^[^ ^17^ ^]^ Copyright 2021, The Royal Society of Chemistry. d) Schematic illustration, e) energy band diagrams and f) corresponding device stability of polymer‐functionalized SnO_2_ as ETLs, for comparison with nontreated SnO_2_ ETLs.^[^ ^33^ ^]^ d–f) Reproduced with permission.^[^ ^33^ ^]^ Copyright 2022, American Chemical Society.

Although size and morphological control approaches have warranted robust crystallization of perovskite layers on top and stable operational stability of PSC devices, interfacial energetics and surface defects still await a synergetic manipulation for maximally enhanced device performance and stability. It was found that by wrapping SnO_2_ NCs with amorphous NbO_*x*_ exterior layer (**Figure** **7**a), the NbO_*x*_‐enveloped ETL exhibited optimized energy‐level alignment with perovskite with smaller charge potential offset (Figure 7b), thereby facilitating carrier extracting while minimizing *V*_oc_ loss.^[^ ^17^ ^]^ Most importantly, unencapsulated PSC devices based on NbO_*x*_‐enveloped SnO_2_ NCs as ETLs exhibited considerably improved device stability under thermal stress conditions (Figure 7c).^[^ ^17^ ^]^ The beneficial effects of SnO_2_ surface wrapping are further corroborated by similar works. Moreover, SnO_2_ NCs as capped with polyacrylamide (PAM) also gave rise to larger perovskite grain size and matched energy‐level alignment with the perovskite layer on top (Figure 7d,e).^[^ ^33^ ^]^ As a result of the ETL modification, unencapsulated solar cell devices based on PAM‐functionalized SnO_2_ ETLs exhibit much enduring performance under 50% relative humidity (RH) by retaining more than 90% of the original efficiency, in contrast with the control devices based on pristine SnO_2_ ETLs (Figure 7f).^[^ ^33^ ^]^

#### 3.1.3 Micro‐ and Nanostructure Designs in SnO_2_ Electron Transport Layers

Although SnO_2_ ETLs possess low‐temperature processability as mentioned in Section 2.3, it can nevertheless be synthesized at high temperatures through hydrothermal methods. Most importantly, in combined with acidic aqueous environment, nanostructured SnO_2_ such as nanorod, nanotube, and nanofiber arrays can be grown.^[^ ^34^ ^]^ Such nanostructured SnO_2_ ETLs possessed controllable area densities or wall thicknesses with well‐defined, and upright structural orientations. When applied in PSCs, devices based on these nanostructured ETLs showed much enhanced PV performance and long‐term stability as compared to the compact ETL‐based control PSCs. Similarly, as aggregated from constituent nanocrystals, SnO_2_ with microsphere shapes with large specific surface areas, high crystallinity, and tunable sizes can be formed,^[^ ^35^ ^]^ resulting in considerable PV performance in PSCs.

#### 3.1.4 Effects of Posttreatments on the Surface Morphology of SnO_2_ Electron Transport Layers

In contrast with the abovementioned approaches that all adopt in situ control over the morphology of SnO_2_ ETLs, posttreatments can also effectively modify the surface morphology of SnO_2_ ETLs. In specific, it was reported that the as‐synthesized SnO_2_ ETLs react with ammonium (NH_3_) that triggered crystal fusion at the surface;^[^ ^36^ ^]^ this surface microstructural modification led to optimized interfacial contact with perovskite layer on top by creating nucleation sites. Based on enhanced interfacial charge transport and suppressed structural defects, PSCs based on the NH_3_‐treated SnO_2_ ETLs showed improved device stability with 86% retained performance after 60 days of aging at room temperature in 40%–50% RH.^[^ ^36^ ^]^ Likewise, posttreatment etching of SnO_2_ ETLs in NH_4_F aqueous solution gave much smoother surface morphology with greater SnO_2_ coverage^[^ ^37^ ^]^ and eventually resulted in higher device PCE and fill factor than the PSCs with pristine SnO_2_ ETLs. Importantly, PSCs based on SnO_2_/NH_4_F ETLs showed >80% retention of initial performance, outperforming the <60% retention on devices with pure SnO_2_ ETL after 30 days of aging in ambient air with 40% RH.^[^ ^37^ ^]^

### 3.2 Surface Physicochemical Modifications

Defects of SnO_2_ thin films greatly influence their electronic properties and the physical quality of perovskite crystallization atop, and the interfaces with perovskite therefore represent active sites that regulate the performance of PSCs.^[^ ^38^, ^39^ ^]^ Passivation of SnO_2_ surface defects commonly adopted surface modification methods including UV/UVO, ionic compounds, carbon derivatives, acids, self‐assembled monolayers (SAM), fullerene derivatives, macromolecular polymers, where a comprehensive summary of device performance and stability of PSCs with surface defect passivation treatments is given in **Table** **2**.

**Table 2:** Various approaches for improving the stability of PSCs by surface defect passivation of SnO_2_ ETLs

Passivation	Device architecture	Stability	Retained PCE	Ref.
UV	ITO/SnO_2_/PVSKa)/PEDOT/MoO_3_/Ag	Unencapsulated, 1 sun, N_2_, 10 h, MPPTb), interface stability	80%	[81]
SVAc)	ITO/SnO_2_/PVSK/Spiro‐OMeTAD/Ag	Unencapsulated, ambient air, 700 h	≈90%	[73]
PSd)	ITO/SnO_2_/Buffer ps/PVSK/Cap ps/Spiro‐OMeTAD/Au	Inner encapsulated, 5 days	97%	[53]
Zwitterionic	FTO/Zw‐SnO_2_/PVSK/HTL/Au	Unencapsulated, 85 °C, RH = 85%, 140 h	93%	[20b]
CIMPIe)	ITO/SnO_2_/PVSK/Spiro‐OMeTAD/Au	Unencapsulated, outdoor, 180 h; 365 nm UV, 100 mW cm^−2^, 2500 h; Unencapsulated, AM1.5G, N_2_, MPPT, 500 h	94%	[77]
			82%	
			80%	
BGClf)	ITO/SnO_2_/BGCl/PVSK/PEAI/MoO_3_/Spiro/Ag	Unencapsulated, 20 °C, RH = 30%, dark, 500 h	95%	[42]
FI‐SnO_2_ g)	FTO/SnO_2_/PVSK/Spiro‐OMeTAD/Au	Unencapsulated, N_2_, 40 °C, 1 sun, 1000 h, mechanically robust	82%	[62]
NbOx	ITO/SnO_2_/NbO_x_/PVSK/Spiro‐OMeTAD/Au	Unencapsulated, N_2_, dark, RT, 2000 h; Unencapsulated, N_2_, 1 sun, 1000 h	95%	[17]
			85%	
KCl	FTO/SnO_2_/PVSK//Spiro‐OMeTAD/Au	Unencapsulated, N_2_, 25 °C, RH = 50%–70%, 720 h	90%	[75]
ImAcH‐Clh)	ITO/SnO_2_/ImAcHCl/PVSK/Spiro‐OMeTAD/Au	Unencapsulated, RT, dark, RH = 40%–60%,35 days	94%	[82]
ADAAi)	ITO/SnO_2_/ADAA/PVSK/Spiro‐OMeTAD/Au	Unencapsulated, RH = 20%–30%, dark, 1200 h; unencapsulated, 60 °C, dark, N_2_, 1000 h; unencapsulated, 1 sun, RT, 1000 h	92.4%	[52]
			81%	
			52%	
KPF_6_	ITO/SnO_2_/KPF_6_/PVSK/Spiro‐OMeTAD/Au	Unencapsulated, RH = 15%–20%, RT, dark, 1440 h; unencapsulated, N_2_, 60 °C, dark, 960 h; unencapsulated, 1 sun, RT, N_2_, 960 h	94.7%	[38]
			80.1%	
			57.2%	
FSAj)	ITO/SnO_2_/FSA/FAPbI_3_/Spiro‐OMeTAD/Au	Unencapsulated, N_2_, AM1.5G, 50–60 °C, MPPT, 1000 h	85%	[80]
I‐SAM	ITO/SnO_2_/I‐SAM/PVSK/HTL/Au	Unencapsulated, 1‐sun continuous illumination, N_2_, RT, MPPT, 3921 h	80%	[49]

a) PVSK: perovskite;

b) MPPT: maximum power point tracking;

c) SVA: solvent vapor annealing;

d) PS: polystyrene;

e) CIMPI: chlorine‐richmixed‐halide perovskite interlayer;

f) BGCl: biguanide hydrochloride;

g) FI‐SnO:FAI‐incorporated SnO_2_;

h) ImAcH‐Cl: 4‐imidazoleacetic acid hydrochloride;

i) ADAA: 1‐adamantaneacetic acid;

j) FSA: formamidine sulfinic acid.

#### 3.2.1 Passivating Oxygen Vacancies at SnO_2_ Surface

**Figure 8:**
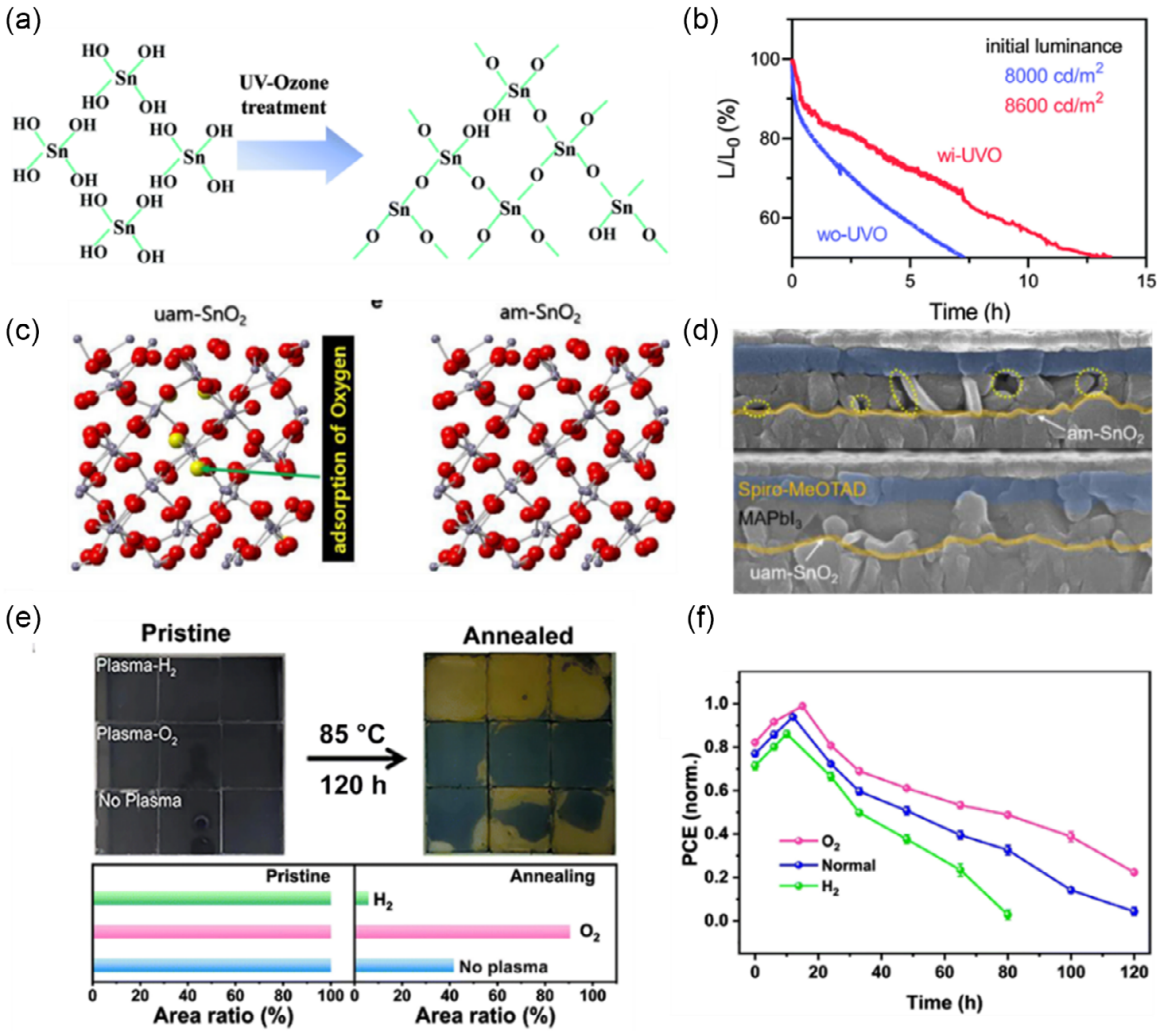
Effects of surface defect annihilation in the device performance stability. a) UVO treatment for elimination of surface hydroxyls from SnO_2_ ETLs.^[^ ^40^ ^]^ b) Stability of light‐emitting devices with or without UVO treatment.^[^ ^40^ ^]^ a,b) Reproduced with permission.^[^ ^40^ ^]^ Copyright 2021, The Royal Society of Chemistry. c) Oxygen vacancy healing by UVO treatment.^[^ ^41^ ^]^ d) Cross‐section SEM images of perovskite/SnO_2_ without (upper) and with (bottom) UVO treatment.^[^ ^41^ ^]^ c,d) Reproduced with permission.^[^ ^41^ ^]^ Copyright 2020, Elsevier. e) Photographs of SnO_2_‐based perovskite films with different plasma treatments before and after thermal stress.^[^ ^9^ ^]^ f) Device stability with SnO_2_ ETLs with plasma treatments in different atmospheric conditions after different thermal stress durations.^[^ ^9^ ^]^ e,f) Reproduced with permission.^[^ ^9^ ^]^ Copyright 2022, The Royal Society of Chemistry.

Among the many passivation strategies, UVO represents one that can effectively remove oxygen vacancies through decomposing hydroxyls, surface dangling bonds, and other organic contaminants from metal oxide surfaces, thereby eliminating carrier recombination centers for robust interfacial stability. The effect of UVO treatment on operation stability of perovskite light‐emitting diodes was previously studied (**Figure** **8**a), and found that the luminance of the fabricated light‐emitting devices exhibited notably enhanced stability with UVO treatment on the SnO_2_ ETLs (Figure 8b).^[^ ^40^ ^]^ With a more in‐depth investigation, it was confirmed that the reduced oxygen vacancies at SnO_2_ surface as functionalized by UVO treatment contribute to a great crystallization quality of perovskite film on top (Figure 8c,d).^[^ ^41^ ^]^ Furthermore, physicochemical relationship between SnO_2_'s surface oxygen vacancies and thermal instability of perovskite thin films was further revealed, as indicated by plasma treatments in different atmospheric gases and a thermal stress at 85 °C for 120 h (Figure 8e).^[^ ^9^ ^]^ As a result of the oxygen vacancy elimination, PSCs based on O_2_‐plasma surface treatment exhibited the greatest stability under imposed thermal stress (Figure 8f).^[^ ^9^ ^]^

**Figure 9:**
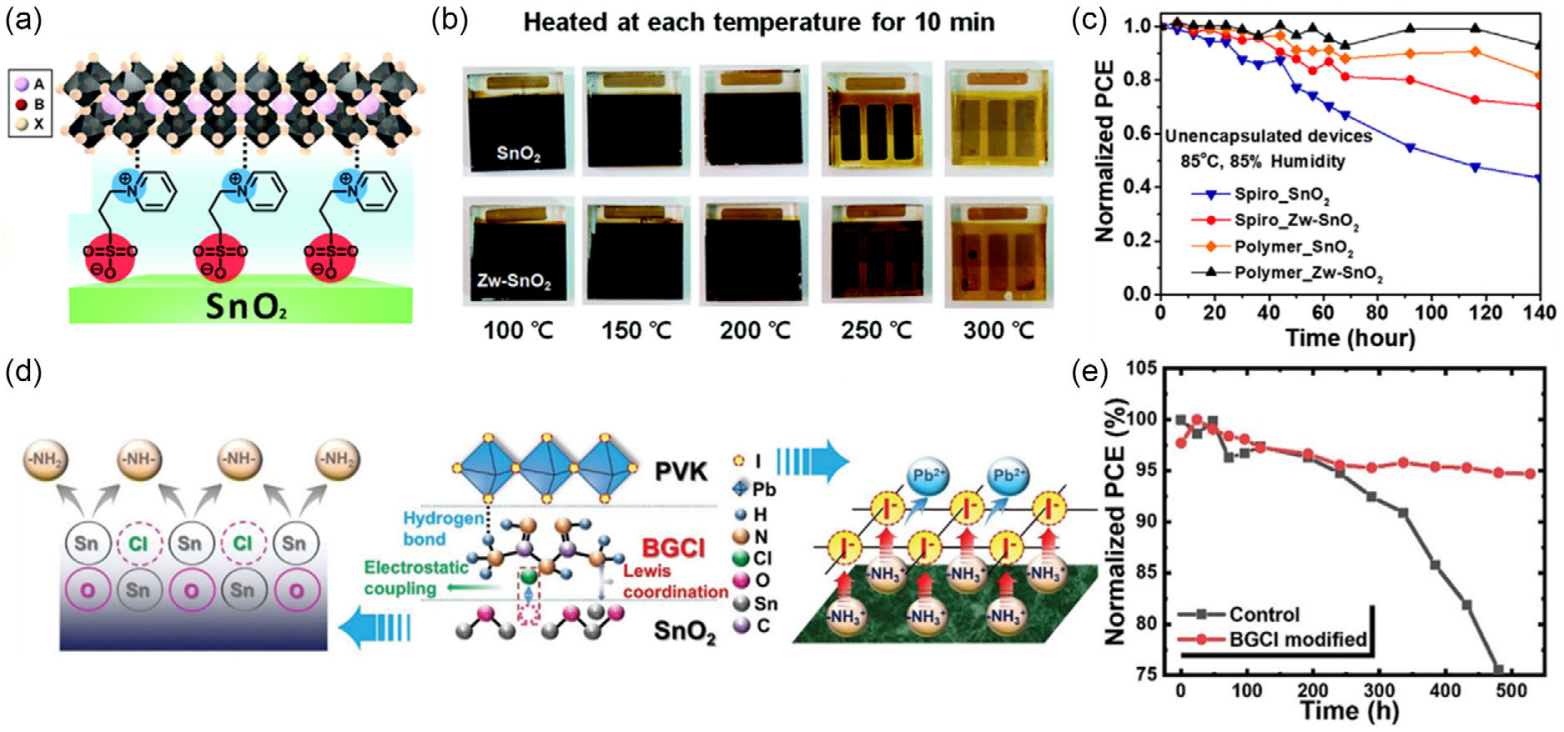
Surface defect passivation of SnO_2_ by dipolar zwitterions and organic halide for stability improvements. a) Schematic depiction of zwitterion passivation of SnO_2_ surface defects.^[20b]^ b) Photographs of perovskite films under thermal stress on different SnO_2_ surface treatments.^[20b]^ c) Device stability of unencapsulated PSCs based on different SnO_2_ treatment and HTLs.^[20b]^ a–c) Reproduced with permission.^[20b]^ Copyright 2018, Royal Society of Chemistry. d) Schematic illustration and interlayer chemistry of BGCl functionalization at the SnO_2_/perovskite interface.^[^ ^42^ ^]^ e) Device stability of PSCs based on pristine and BGCl‐treated SnO_2_ ETLs.^[^ ^42^ ^]^ d,e) Reproduced with permission.^[^ ^42^ ^]^ Copyright 2022, Wiley‐VCH GmbH.

In terms of chemical passivation of SnO_2_ surface defects, passivation molecules at the SnO_2_/perovskite interfaces should have both electronegative and electropositive functional groups to passivate the undercoordinated Sn (electron‐poor) and O (electron‐rich) atoms. After passivation, surface dangling bonds associated with these undercoordinated surface atoms as recombination centers are eliminated due to the formation of new chemical bonds.^[10a]^ To meet such requirements of surface passivation, zwitterions are the ideal choices as passivation agents. Zwitterion—3‐(1‐pyridinio)‐1‐propanesulfonate was previously used to modify SnO_2_ ETL to improve the thermal stability of solar cell device.^[20b]^ The zwitterionic compound led to the formation of interfacial dipole (**Figure** **9**a) and a changed work function of SnO_2_ ETL, thus preventing reversed electron transfer and inhibiting charge recombination. Furthermore, due to the positively charged ions, zwitterion passivated Pb/I antisite defects within perovskite lattice, thereby improving thermal stabilities of perovskite films (Figure 9b) and solar cell devices under double 85 test conditions (Figure 9c).^[20b]^ Likewise, bifunctional ionic additive—biguanide hydrochloride (BGCl) was applied at the interface of SnO_2_/perovskite to form hydrogen bonding with halides at perovskite's bottom surface while forming electrostatic and Lewis coordination with the underlying SnO_2_ ETL (Figure 9d), thus passivating the interfacial defects in a holistic format. As a result of the interface modifications, BGCl‐treated solar cell devices exhibit a remarkable 24.4% device efficiency. Without encapsulation, BGCl‐treated devices only sustained less than 5% loss of efficiency after 500 h of aging under ambient conditions with 30% RH at 20 °C (Figure 9e).^[^ ^42^ ^]^

**Figure 10:**
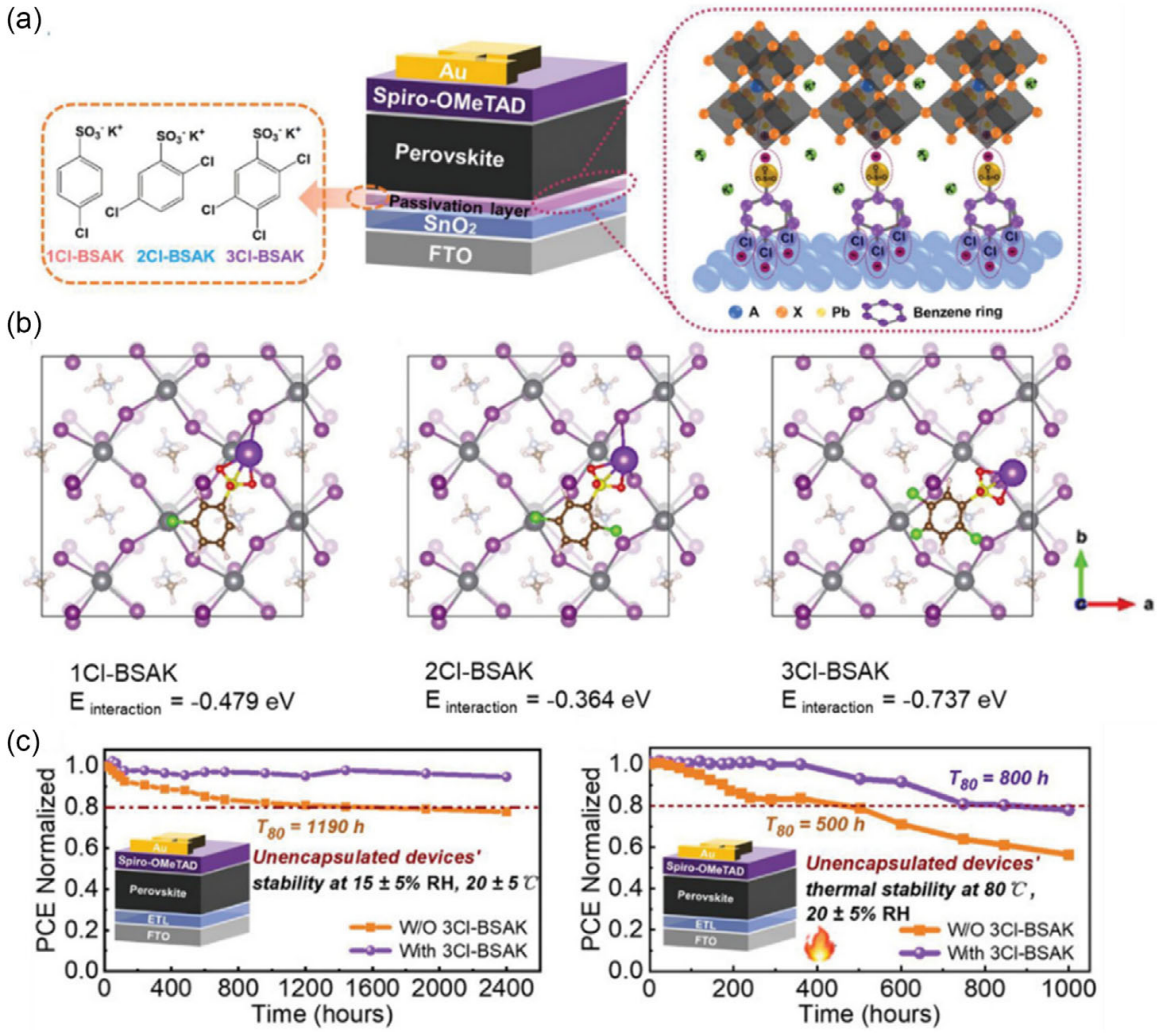
a) Device and mechanistic schematics of chlorobenzene sulfonic potassium salts in passivating the buried interfaces of PSCs.^[^ ^43^ ^]^ b) Theoretical models of perovskite‐BSAK interactions used in first‐principles DFT calculations.^[^ ^43^ ^]^ c) Stability tests of unencapsulated devices at room temperatures (left) and elevated temperature conditions (right).^[^ ^43^ ^]^ a–c) Reproduced with permission.^[^ ^43^ ^]^ Copyright 2022, Wiley‐VCH GmbH.

The effects of interfacial passivator in defect suppression can be further tuned by tailoring the number and position of functional groups on organic ring structures. It was previously demonstrated that chlorobenzene sulfonic potassium (Cl‐BSAK) salts can fill oxygen vacancies on the SnO_2_ surface via coordination bonds between Cl and Sn (**Figure** **10**a).^[^ ^43^ ^]^ In contrast, first‐principles density functional theory (DFT) calculations revealed the inclined interaction between sulfonate group and perovskite, where most stable configuration was achieved by 3Cl‐BSAK on perovskite surface (Figure 10b).^[^ ^43^ ^]^ Furthermore, it was found that 3Cl‐BSAK with the greatest number of Cl was conducive to the greatest performance enhancement of PSCs, in which unencapsulated devices based on 3Cl‐BSAK treatment exhibited more than 95% retention of original efficiency after 2400 h of aging in dry environment and 80% retention of initial performance after 800 h of thermal stress at 80 °C (Figure 10c).^[^ ^43^ ^]^

**Figure 11:**
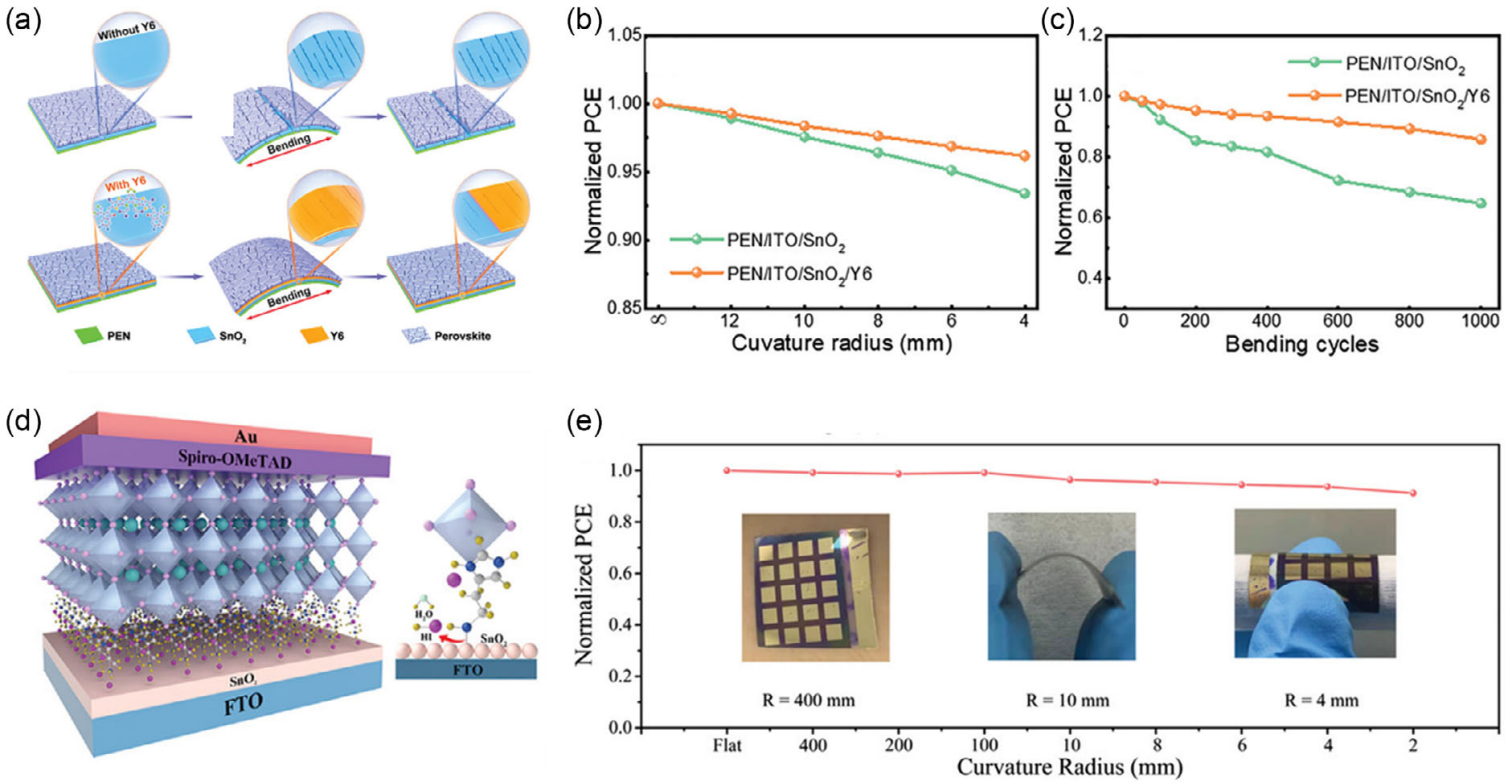
Additive‐functionalized SnO_2_ ETLs for stable flexible PSCs. a) Schematic illustration of Y6 functionalization in SnO_2_ for mechanically durable flexible PSCs.^[^ ^45^ ^]^ b,c) Device stability of flexible PSCs based on pristine and Y6‐treated SnO_2_ ETLs at different bending curvatures (b) and at 8‐mm radius for different cycles (c).^[^ ^45^ ^]^ a–c) Reproduced with permission.^[^ ^45^ ^]^ Copyright 2022, Wiley‐VCH GmbH. d) Schematic illustration of PSC device with HADI treatment on the SnO_2_ ETLs.^[^ ^46^ ^]^ e) Stability test of flexible PSCs at different bending curvature radius. Inset: photographs of flexible PSCs under bending conditions.^[^ ^46^ ^]^ d,e) Reproduced with permission.^[^ ^46^ ^]^ Copyright 2022, Wiley‐VCH.

Due to the large deformation of substrates during thermal annealing, flexible PSCs suffer from large interfacial residual stress issues that largely plague the operational stabilities of PSCs under bending or illumination conditions. From mechanistic perspective, the large interfacial residual stress usually stems from interfacial defects or discrepant thermal expansivity between ETLs and perovskites.^[^ ^44^ ^]^ Therefore, it is imperative to develop modification strategies that can achieve defect passivation of SnO_2_ ETLs to resolve the instability issues in flexible PSCs. Non‐fullerene acceptor molecule Y6 (fused dithienothiophen[3,2‐b]‐pyrrolobenzothiadiazole derivative) was previously used as a buffer layer in SnO_2_ ETLs (**Figure** **11**a).^[^ ^45^ ^]^ The Y6 treatment greatly resulted in relaxed interfacial stress, and the as‐fabricated flexible PSCs showed evidently improved mechanical durability at different bending radius and bending cycles (Figure 11b,c).^[^ ^45^ ^]^ Similarly, multifunctional histamine diiodide (HADI) additive was applied to cure the defects at the buried interface of SnO_2_/perovskite (Figure 11d), forming interlayer bridging structures that refine charge transfer and energy band alignments.^[^ ^46^ ^]^ Consequently, flexible PSCs with HADI‐treated SnO_2_ ETLs displayed exceptional mechanical stability upon different bending curvature radii (Figure 11e), thus suggesting the transferable efficacy of multifunctional ionic additives in improving both rigid‐based and flexible PSCs.^[^ ^46^ ^]^

#### 3.2.2 Inhibiting Surface Hydroxyls of SnO_2_ Electron Transport Layers

**Figure 12:**
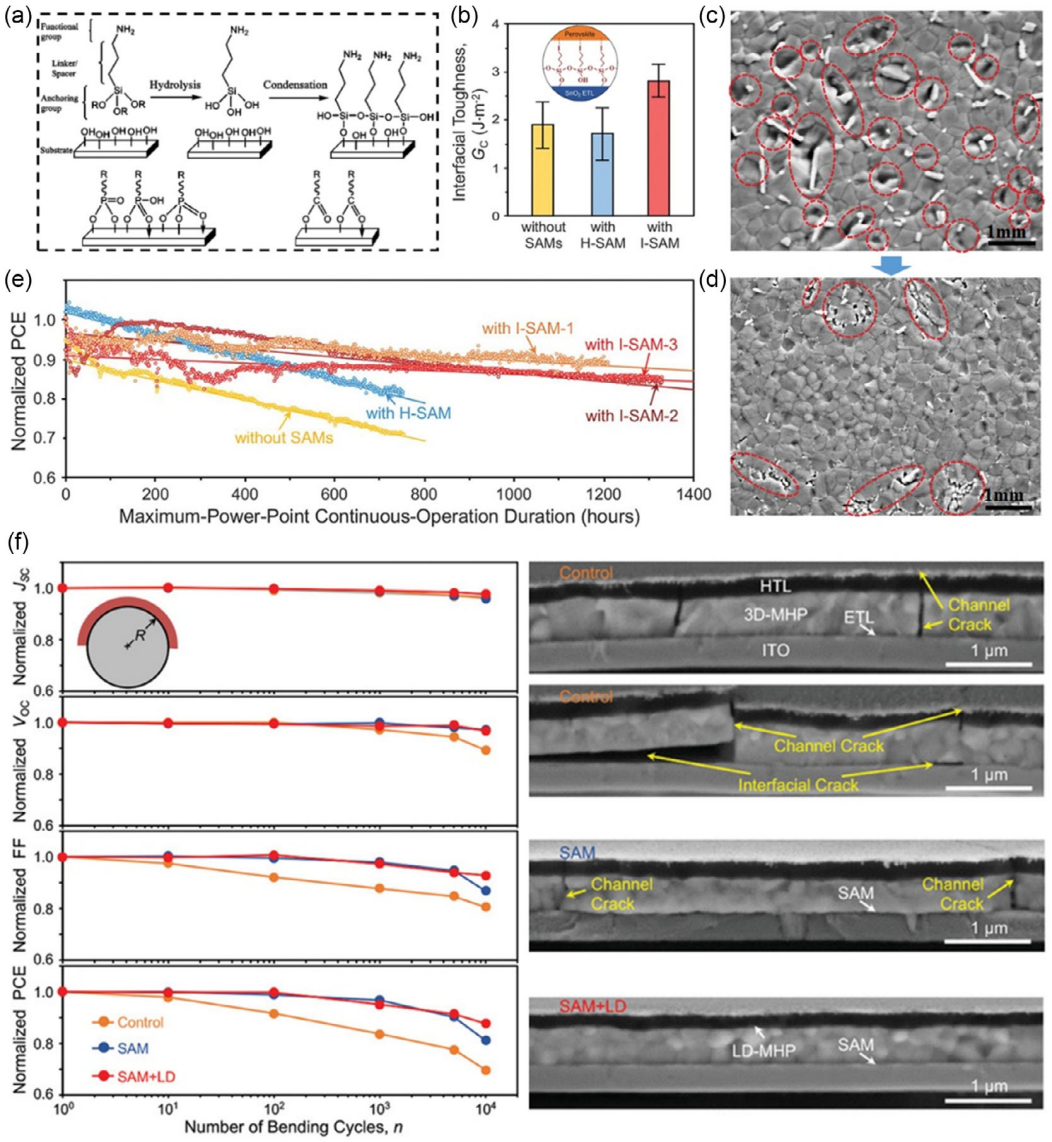
SAMs in achieving surface defect passivation of SnO_2_ ETL. a) Schematic diagram of synthetic process of SAMs.^[^ ^48^ ^]^ Reproduced with permission.^[^ ^48^ ^]^ Copyright 2020, Wiley‐VCH. b) Mechanical toughness of SnO_2_/perovskite interfaces without and with SAMs functionalization. c,d) SEM images of perovskite surfaces as delaminated from substrates without SAM treatment (c) and with iodine‐terminated SAM (d). e) Operational stability of PSCs without and with SAM treatments under continuous one‐sun illumination and maximum power point (MPP) tracking.^[^ ^49^ ^]^ b–e) Reproduced with permission.^[^ ^49^ ^]^ Copyright 2021, American Association for the Advancement of Science. f) Mechanical reliability of flexible PSCs under bending stress. Left: device stability as function of bending cycle with at a radius of 5 mm. Right: cross‐section SEM images of flexible devices with and without SAM at the SnO_2_/perovskite interface after the 10 000‐cycle bending. Reproduced with permission.^[^ ^50^ ^]^ Copyright 2022, Wiley‐VCH.

As pointed out in previous context, surface hydroxyls of SnO_2_ ETLs play a significant role in causing the instabilities of perovskite layers and solar cell devices, where the existence of hydroxyls not only induce intrinsic defects of SnO_2_ ETLs but also negatively impact the buried interface between SnO_2_ and perovskites. To mitigate the defective interface as a whole, researchers have come up with novel strategies that used SAMs to optimize the interfaces.^[^ ^47^ ^]^ A typical SAM molecule is structurally composed of three parts: anchoring group, interterminal group, and terminal group (**Figure** **12**a).^[^ ^48^ ^]^ To react with surface hydroxyls, anchoring groups of SAMs (typically phosphonic, hydroxyls, and carboxylic acid groups) first bind with the hydroxyls at SnO_2_ surface through condensation, whereas terminal groups of SAMs can then have the potentials to fill the halide vacancies of perovskite bottom surface, thus modifying the buried interface. A concept of “molecular glue” at the SnO_2_/perovskite interface was previously proposed by adopting 3‐iodopropyl trimethoxysilane (Si(OCH_3_)_3_CH_2_)_3_I, I‐SAM) as molecular treatment to enhance adhesion toughness between SnO_2_ and perovskite, as shown in Figure 12b.^[^ ^49^ ^]^ The mechanical strength of I‐SAM‐treated SnO_2_/perovskite interface could be further illustrated by the much less voids/cracks formed upon perovskite film delamination from the substrate (Figure 12d), as compared to the fractured perovskite film without SAM functionalization with rampant morphological defects (Figure 12c).^[^ ^49^ ^]^ The increased interfacial mechanical stability resulted in profound operational stabilities of I‐SAM‐treated PSCs under continuous illumination, in comparison with the device counterparts treated with H‐terminated SAM (H‐SAM) and control devices without SAM treatments (Figure 12e).^[^ ^49^ ^]^ The observed device stability brought by interfacial I‐SAM again corroborates the importance of multifunctional treatments that not only chemically passivate SnO_2_ ETLs but also modify the bottom surfaces of perovskite layers.

Particularly, the application of SAMs at the ETL/perovskite interface finds the best application scenario in flexible PSCs. As demonstrated in the above context, SAM can formidably enhance interfacial toughness of SnO_2_/perovskite junction. And when applied in flexible PSCs, it was shown that their performance stability under bending stress was greatly improved (Figure 12f, left).^[^ ^50^ ^]^ In detail, SAM‐functionalized flexible PSCs with optimized upper surface LD structural engineering yielded 88% retention of original performance after 10 000 cycles at a bending radius of 5 mm, while showing T_90_ operational lifetime of 1000 h under continuous one‐sun illumination, which remarkably outperformed the flexible PSC counterparts with untreated SnO_2_/perovskite interfaces that exhibited T_80_ lifetime of only 600 h, currently topping the operational performance stability among all flexible PSCs reported.^[^ ^50^ ^]^ The corresponding cross‐section morphology of untreated flexible PSCs showed omnipresent channel and interfacial cracks that were distributed after the bending stress, whereas SAM + LD devices were free of such morphological degradation (Figure 12f, right). By comparing the abovementioned modifications in flexible PSCs, one can infer on the much greater efficacy of interfacial SAMs in curing the surface defects of SnO_2_, and thus the enhancements in mechanical and operational stabilities of flexible PSCs than just the ionic additives.

#### 3.2.3 Enhancing Surface Adhesion of SnO_2_ Electron Transport Layers

**Figure 13:**
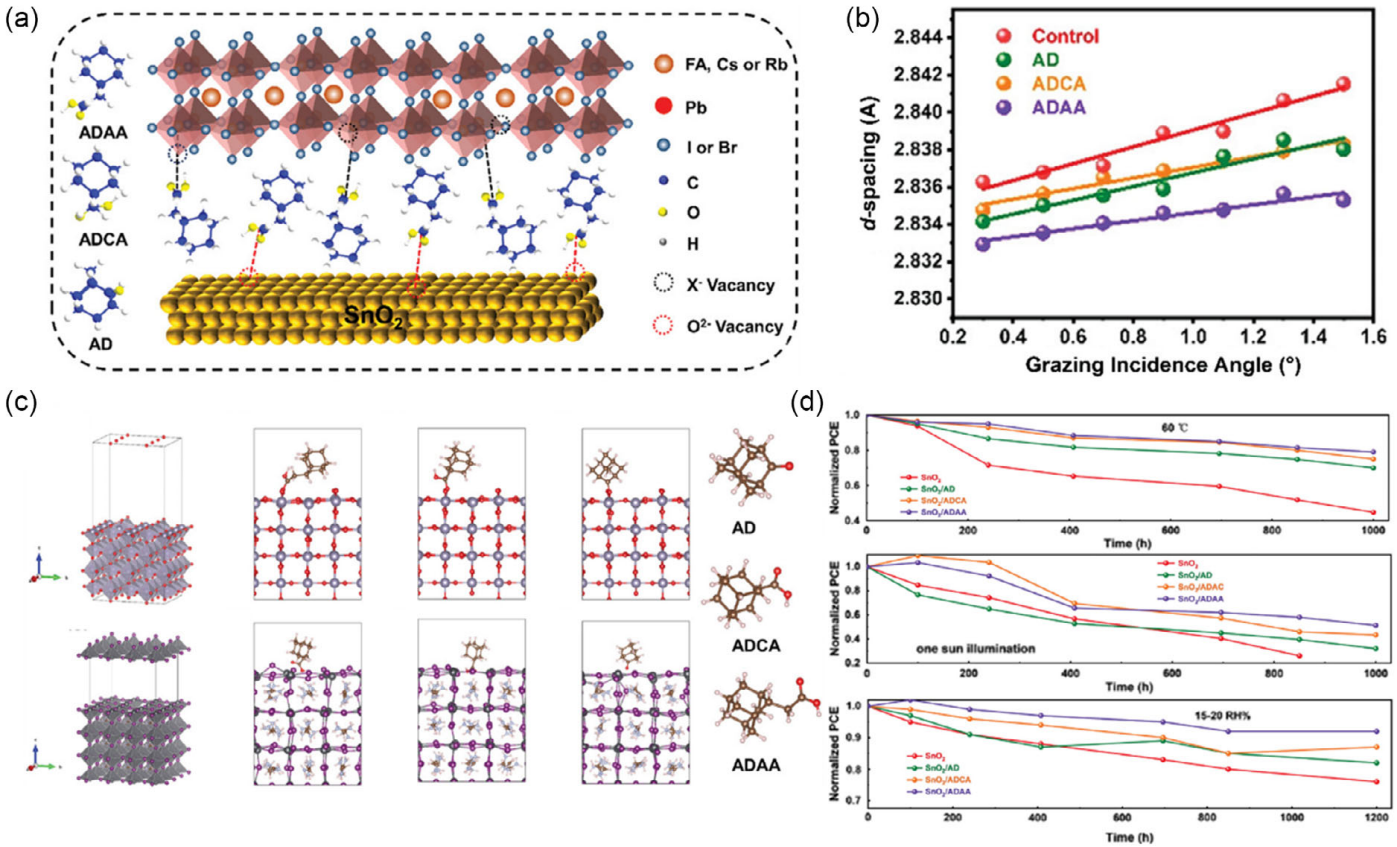
Strain relaxation of SnO_2_/perovskite interfaces for stable device performance. a) Schematic illustration of SnO_2_/perovskite interfaces as passivated by adamantane derivative molecules.^[^ ^52^ ^]^ b) Grazing‐incidence angle‐dependent lattice spacings of perovskite films on SnO_2_ ETLs with different molecular interfacial treatments.^[^ ^52^ ^]^ c) Theoretical models of SnO_2_ and perovskite surfaces with different adamantane molecular passivators.^[^ ^52^ ^]^ d) Stability tests of control and target devices with molecular treatments under 60 °C thermal stress (top), continuous one‐sun illumination (middle), and 15%–20% RH conditions (bottom).^[^ ^52^ ^]^ a–d) Reproduced with permission.^[^ ^52^ ^]^ Copyright 2022, Wiley‐VCH.

Although we have shown that extensive efforts have successfully passivated surface defects in SnO_2_ ETLs, the stability of PSCs nevertheless also depends on the residual stress sustained by perovskite layers.^[^ ^51^ ^]^ From fundamental perspectives, residual stress at the interface arises from discrepant thermal expansivity between ETL and perovskite film after thermal annealing.^[51c]^ Thermally induced tensile stress in the perovskite film weakens chemical bonds, reduces the formation energy of defects, and reduces the activation energy of ion migration, which therefore accelerates the degradation of perovskite film under illumination and electrical bias conditions.^[^ ^9^ ^]^ In this regard, adamantane derivative molecules (AD, ADCA, and ADAA) containing carbonyl (C = O) functional groups were used to modify the SnO_2_ ETL/perovskite contact for passivating the interfacial defects, while releasing the residual stress therein. The passivation molecule coordinates with the oxygen vacancy defects in SnO_2_ while simultaneously binding with the undercoordinated Pb in perovskite (**Figure** **13**a).^[^ ^52^ ^]^ In contrast, functionalized SnO_2_ ETLs with the molecular treatments led to notable relief of residual stress in perovskite thin films (Figure 13b).^[^ ^52^ ^]^ Among the three molecular modifiers, theoretical modeling further confirms the vital roles of steric hindrance in affecting the interfacial stress relaxation and thus the integral stability of PSC devices (Figure 13c), where the stabilizing effects exhibit an order of ADAA > ADCA > AD on unencapsulated devices under thermal stress, continuous one‐sun illumination and ambient conditions (Figure 13d).^[^ ^52^ ^]^ The results indicate that unlike chemical modification of SnO_2_/perovskite buried interface, the ordered arrangement of passivation molecules according to their mutual steric hindrance is also an important factor for consideration in regulating interfacial residual stress.

**Figure 14:**
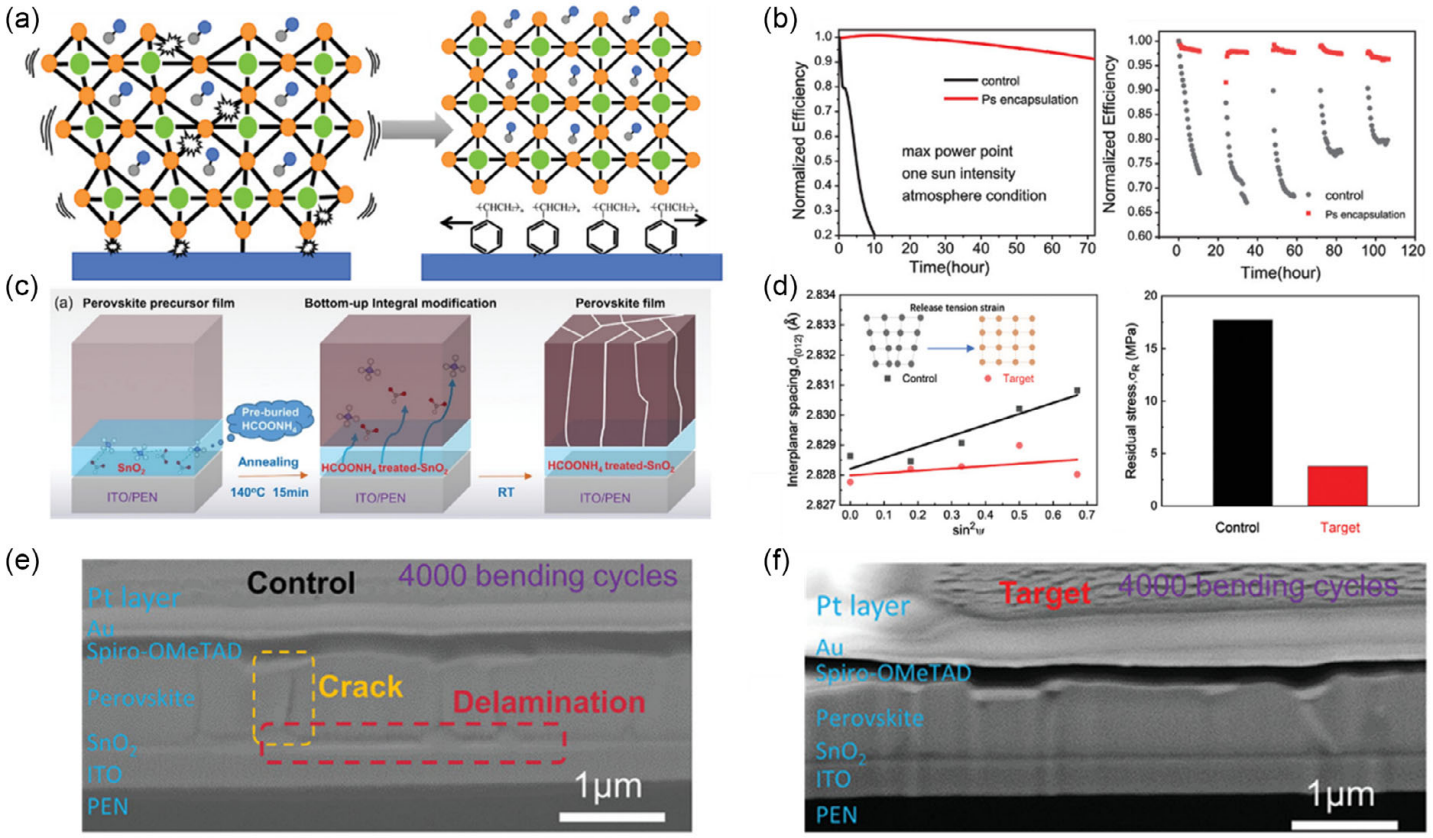
Residual stress release in perovskite layers via interfacial modifications. a) Schematic depiction of stress release of SnO_2_/perovskite interface by PS layer.^[^ ^53^ ^]^ b) Operational stability under continuous one‐sun illumination (left) and illumination–dark cycle stability (right) of PSC devices without and with PS buffer layer.^[^ ^53^ ^]^ a,b) Reproduced with permission.^[^ ^53^ ^]^ Copyright 2019, Wiley‐VCH. c) Schematic illustration of preburied additive for cross‐layer treatment of SnO_2_/perovskite interface.^[^ ^44^ ^]^ d) *d*‐spacing‐sin^2^ψ method for calculating the residual stress of perovskite films with and without NH_4_COOH preburied additive.^[^ ^44^ ^]^ e,f) Cross‐section SEM images of PSC devices after 4000 bending cycles with (e) and without (f) preburied NH_4_COOH in SnO_2_ ETLs.^[^ ^44^ ^]^ c–f) Reproduced with permission.^[^ ^44^ ^]^ Copyright 2022, Wiley‐VCH.

Instead of regulating the interfacial residual stress through organic molecule's steric hindrance, polystyrene (PS) was introduced into PSCs as a buffer layer between SnO_2_ ETL and perovskite film, as shown in **Figure** **14**a.^[^ ^53^ ^]^ Due to the soft polymer nature, PS formed continuous film and properly alleviated the interfacial strain between SnO_2_ ETL and perovskite film by reducing the modulus mismatch, thereby improving the structural integrity of perovskite lattice. Consequently, PSCs with PS interfacial treatment showed greatly enhanced operation stability under continual one‐sun illumination in ambient atmosphere, as well as illumination–dark cycle stability for more than 100 h (Figure 14b).^[^ ^53^ ^]^ Instead of adopting the mindset of interfacial buffer layer, an idea of implanting volatile organic additive—ammonium formate (NH_4_COOH) within SnO_2_ ETL was previously devised, where NH_4_COOH vaporized during perovskite film annealing to realize an in situ and integral modification of SnO_2_ ETL, perovskite layer and their interface (Figure 14c), thus achieving a formidably released interfacial stress (Figure 14d).^[^ ^44^ ^]^ Most profoundly, perovskite films on flexible substrates functionalized with such preburied NH_4_COOH additive exhibited minimum morphological degradation (Figure 14f), in contrast to the untreated films that showed obvious cracks and delamination after 4000 cycles of mechanical bending (Figure 14e), much alike to the strengthening effects of I‐SAM on the interfacial toughness as discussed above.^[^ ^44^ ^]^ However, differentiation needs to be made between the treatments on SnO_2_ ETLs by halide‐containing molecules (e.g., I‐SAM) and the ammonium‐based volatile additives (e.g., NH_4_COOH) as owing to their different working mechanisms of interfacial modifications. In specific, previous research revealed that light‐induced performance degradation of PSCs mainly arose from the generation of iodide interstitials (I_I_^−^ and I_I_^+^) at the perovskite/charge transport layer interfaces; where electric‐bias induced degradation also originated from the interaction between I_I_^−^ and hole carriers transferred from ETL.^[^ ^54^ ^]^ Such device degradations as dictated by halide‐defect formation and evolution behaviors thus signify discrepant passivation effects by different chemical treatments of SnO_2_ ETLs.

### 3.3 Surface Composite‐Structure Designs

From the energy level and electronic property perspectives, ETLs need to have high electron mobility, fine energy‐level alignment, and good surface coverage of TCO substrates. Also, an optimized interfacial energy‐level structure is exceptionally important to good charge extraction and transport, a basic requirement for reduced interfacial charge accumulation for good device stability.^[^ ^55^, ^56^ ^]^ To meet these material characteristics, a meticulously composited ETL structure is demanded, such as double ETLs and planar/mesoporous composited structures, where **Table** **3** explicates a comprehensive summary of device performance and stability based on composite‐structured ETL designs from recent works.

**Table 3:** Various approaches to enhance the stability of perovskite solar cells with double electron layers

Double ETLs	Device architecture	Stability	Retained PCE	Ref.
TiO_2_/SnO_2_	TCO/TiO_2_/SnO_2_/PVSK/HTL/Au	Unencapsulated, air, 80 °C, RH = 30%–35%, 200 h	95%	[39]
SnO_2_/TiO_2_	ITO/SnO_2_/TiO_2_ /PVSK/Spiro‐OMeTAD/Au	Unencapsulated, N_2_, no degradation after 49 days	\	[56b]
TiO_2_/SnO_2_	FTO/TiO_2_/SnO_2_/PVSK/Spiro‐OMeTAD/Au	Unencapsulated, N_2_, RH = 0%, RT, AM1.5G, MPPT,^a^) 1000 h	97%	[79]
SnO_2_+NH_4_Cl/SnO_2_+NH_4_Cl	ITO/B‐SnO_2_/Perovskite/PEAI/Spiro‐OMeTAD/Au	Unencapsulated, argon, RT, 90 days; Unencapsulated, AM1.5G, RT, 100 h	97.1%	[58]
			86.5%	
In_2_O_3_/SnO_2_	ITO/In_2_O_3_/SnO_2_ /PVSK/Spiro‐OMeTAD/Au	Unencapsulated, N_2_, 25 °C, 80 days; Unencapsulated, 1 sun, 180 h; Unencapsulated, RH = 75%, 120 h	97.5%	[55]
			91%	
			80%	
SnO_2_/MgO	ITO/SnO_2_/MgOPVSK/Spiro‐OMeTAD/Au	Unencapsulated, RH < 30%, STC,b) dark, 107 days; AM1.5G, 100 mW cm^−2^, MPPT, 107 days	68%	[83]
			67.4%	
SnO_2_/DPC_60_ c)	ITO/SnO_2_/DPC_60_ PVSK/HTL/Au	Unencapsulated, N_2_, 1 sun, 55 ± 5 °C, MPPT, 200 h; Unencapsulated, RH = 35 ± 5%, 55 ± 5 °C, 30 days	82%	[84]
			93%	

a) MPPT: maximum power point tracking;

b) STC: standard test conditions (i.e., irradiance of 100 mW cm^−2^, AM1.5G spectrum, at 25 °C);

c) DPC_60_: 2,5‐diphenyl C_60_ fulleropyrrolidine.

#### 3.3.1 Suppressing Interfacial Charge Accumulation by Double Electron Transport Layers

**Figure 15:**
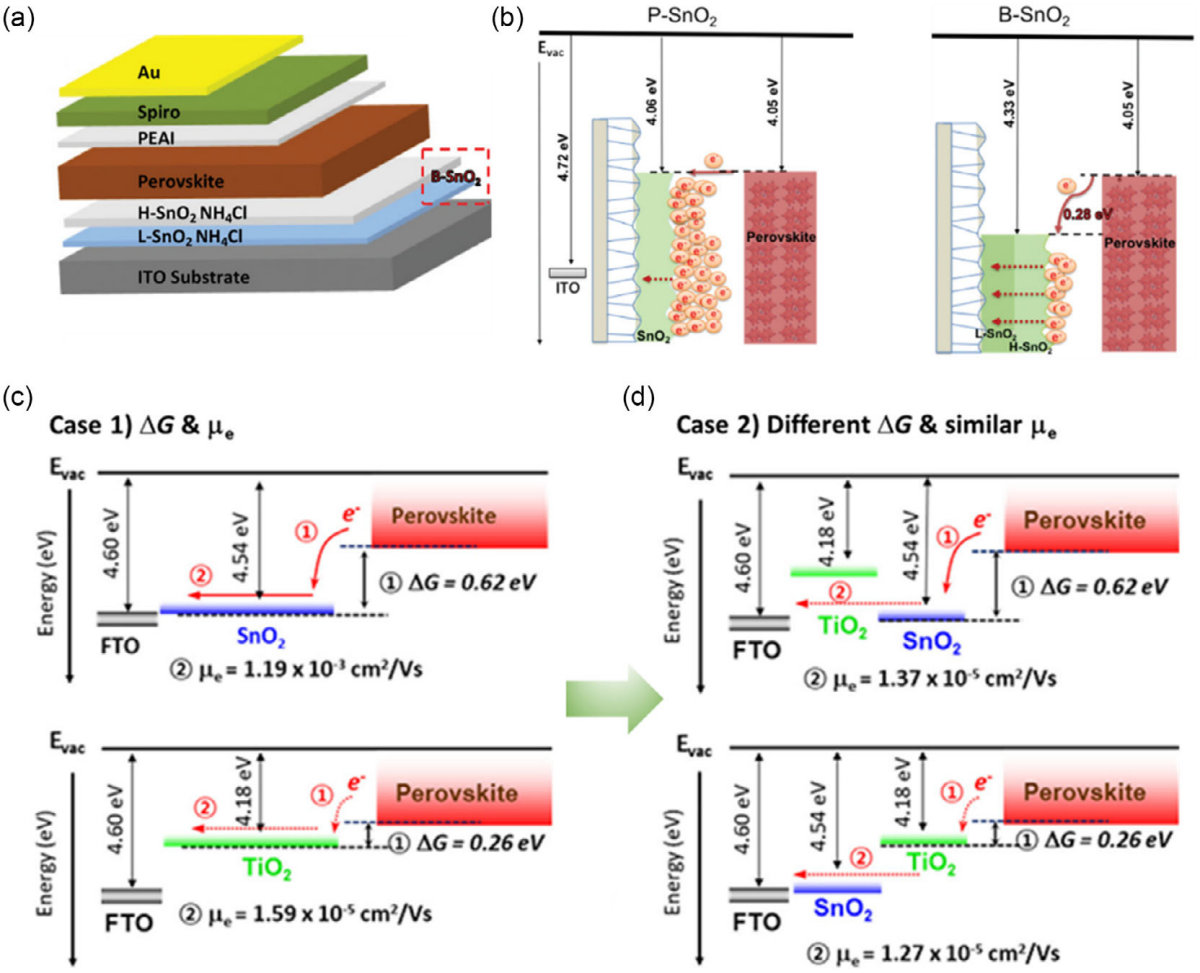
Double ETLs in PSCs for regulating energy‐level alignments. a) Device structure of PSCs employing H‐SnO_2_ and L‐SnO_2_ double ETLs with NH_4_Cl additive.^[^ ^58^ ^]^ b) Band alignment schematics of perovskite layers on single (left) and double (right) SnO_2_ ETLs.^[^ ^58^ ^]^ a,b) Reproduced with permission.^[^ ^58^ ^]^ Copyright 2020, Wiley‐VCH. c,d) Schematic diagrams of interfacial ΔG and *μ*_e_ with SnO_2_ (top) and TiO_2_ (bottom) being the single ETLs (c), and the arrangements of TiO_2_/SnO_2_ (top) and SnO_2_/TiO_2_ (bottom) being the double ETLs (d).^[^ ^59^ ^]^ c,d) Reproduced with permission.^[^ ^59^ ^]^ Copyright 2017, American Chemical Society.

Interfacial charge accumulation is detrimental to the stabilities of PSCs by accelerating ion migrations, thus triggering lattice decomposition of perovskite materials.^[^ ^57^ ^]^ As a typical example, it was previously shown that the energy levels of SnO_2_ ETLs can be effectively tuned by composing low‐amount and high‐amount NH_4_Cl‐doped composite SnO_2_ ETLs (**Figure** **15**a),^[^ ^58^ ^]^ in which the charge potential difference between composite SnO_2_ ETLs and perovskite layers can be properly enlarged to promote light‐induced charge extraction from the latter, thus facilitating the subsequent carrier transport to TCO substrate and avoiding charge accumulation (Figure 15b).^[^ ^58^ ^]^ A deeper mechanistic picture of the principles was further built, which needs to be followed for designing double ETLs, where composite order of TiO_2_ and SnO_2_ need to be scrutinized in terms of their free energy differences (Δ*G*) with perovskites and the resultant electron mobility within the ETLs (Figure 15c,d).^[^ ^59^ ^]^ Moreover, composite‐structure designs featured with SnO_2_‐based dual ETLs in p–i–n structured PSCs have also been reported, where C_60_/SnO_2_ NCs and PCBM/SnO_2_ NCs double ETLs were employed on top of perovskite layers to enhance electron extraction. These composite structures, as compared to pristine C_60_ and PCBM ETLs, showed much more cascaded energy alignments with perovskites, thus resulting in mitigated hysteresis, overall performance, and long‐term stability of PSCs.^[^ ^18^, ^60^ ^]^ Based on these designing principles, researchers have constructed double ETLs with largely enhanced operational stabilities of the corresponding PSCs under different environmental stresses, whose performance and stability profiles are summarized in Table 3.

#### 3.3.2 Enhancing Mechanical Stability by Interpenetrative Electron Transport Layers

**Figure 16:**
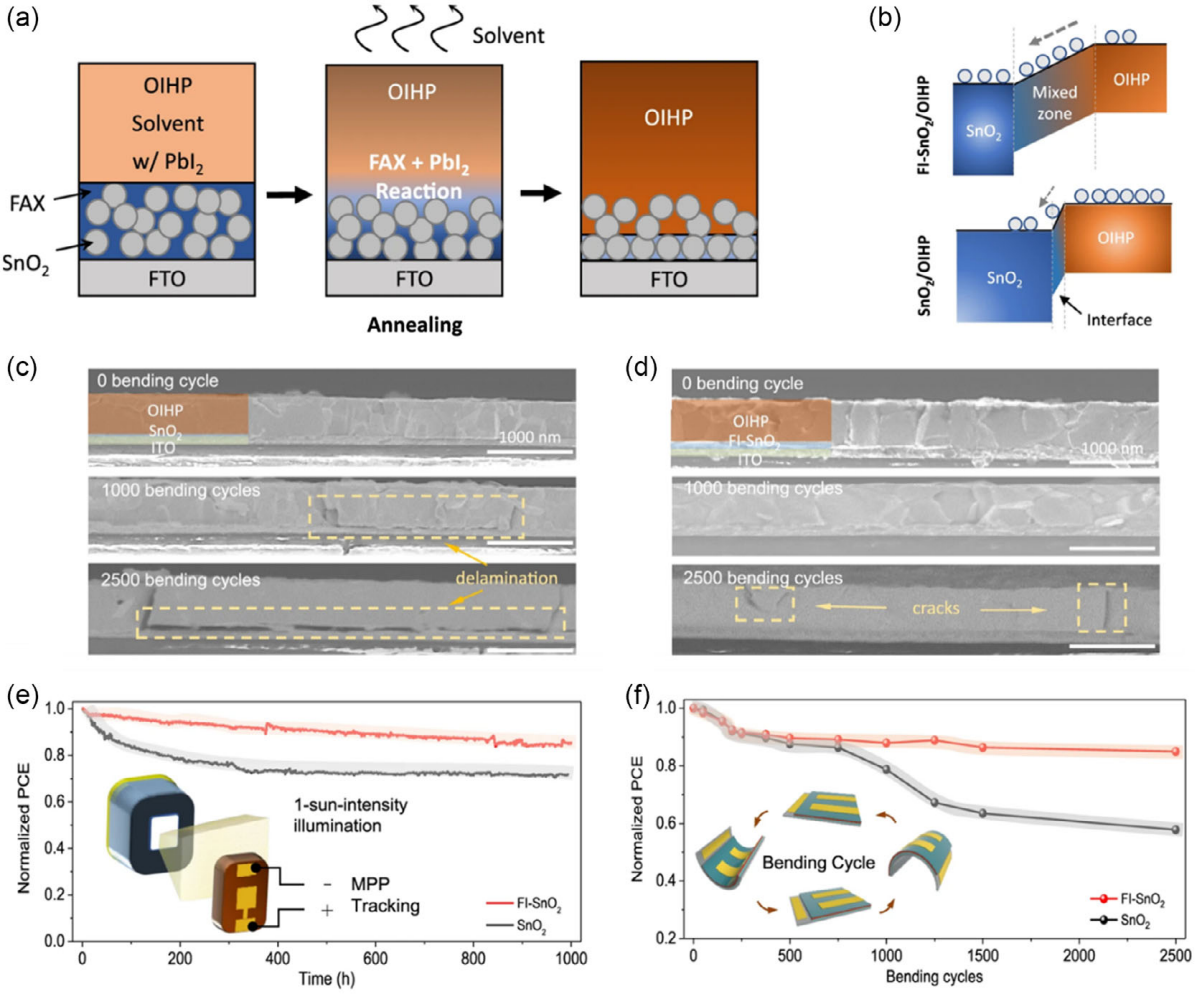
FAI‐modified SnO_2_ ETLs for interpenetrating interfaces in stable PSCs. a) Schematic illustrations of the preparation method of interpenetrative SnO_2_ ETL and b) energy‐level cascading between perovskite and pristine/interpenetrative SnO_2_ ETLs.^[^ ^62^ ^]^ c) Cross‐section SEM images of perovskite films deposited on pristine and d) interpenetrative SnO_2_ ETLs before and after mechanical bending.^[^ ^62^ ^]^ e) Stability tests of rigid PSCs under continuous one‐sun illumination and f) flexible PSCs with mechanical bending.^[^ ^62^ ^]^ a–f) Reproduced with permission.^[^ ^62^ ^]^ Copyright 2021, Springer Nature.

In addition to the interfacial charge accumulation, device stability is also dependent on the detailed surface morphological properties of ETL composites. Different from the mesoporous ETL structures, where the porosity of ETL materials such as TiO_2_ is realized through thermal sintering the solution‐deposited nanoparticle precursors,^[^ ^61^ ^]^ interpenetrative SnO_2_ ETLs can also be achieved by the chemical reaction between the implanted precursor dopants therein and perovskite layers on the top. It was previously found that porous and interpenetrating SnO_2_ ETL as generated from the evaporation of preburied formamidinium iodide (FAI) (**Figure** **16**a) could led to a more cascaded energy‐level structure between SnO_2_ and perovskites (Figure 16b).^[^ ^62^ ^]^ Also, the effects of the interpenetrating SnO_2_/perovskite interface can profoundly enhance physical adhesion of perovskite film on the SnO_2_ ETL substrate, as manifested in the phenomenally enhanced mechanical strength that is free of interfacial delamination after bending stress (Figure 16d), in comparison with the perovskite film without the SnO_2_ porosity treatment (Figure 16c).^[^ ^62^ ^]^ In the end, PSCs based on interpenetrating SnO_2_/perovskite interfacial design exhibited greater operational stability under continuous one‐sun illumination as imparted from maximum power point (MPP) tracking (Figure 16e) and mechanical durability of flexible PSCs with bending stress conditions (Figure 16f).^[^ ^62^ ^]^

## 4 Summary and Outlook

This review analyzes the interconnection between SnO_2_ ETLs and device stability as the focal point, with topics ranging from material properties and film formation of SnO_2_ ETLs to the passivation and modification of interfacial defects. Summarizing the aforementioned works, degradations to the PV performance of PSCs related to the SnO_2_ interface can be deduced into three categories: 1) surface defects of SnO_2_ ETLs that facilitate the oxygen diffusion in perovskite layers that weaken the thermal/chemical stabilities of perovskite materials; 2) charge carrier accumulation and the related ion migration due to interfacial energy level misalignment, causing device instability under illumination and electrically biased conditions; and 3) interfacial stress induced by the lattice mismatch between perovskite and SnO_2_ ETLs, causing weakened mechanical durability and performance degradation that are most significant in flexible PSCs. Accordingly, three manipulation strategies imposed on SnO_2_ surface to tailor its physical and electronic compatibility in PSCs—surface morphological control, defect passivation, and composite‐structure designs, have been utilized to improve the PSCs’ PV performance and operational stability. Several substantiated conclusions are deduced as: 1) Surface morphological profiles of SnO_2_ ETLs directly affect the film growth and crystallization quality of perovskite layers atop. 2) Hydroxyl groups and the existence of dangling bonds on the SnO_2_ surface lead to reduced formation energy of oxygen vacancies that will accelerate deprotonation process of perovskite lattice under illumination and elevated temperature conditions, thus affecting stabilities under light and thermal stresses. 3) Composite‐structure designs are conducive to reduced energy band offsets between ETLs and perovskites, thus inhibiting interfacial carrier accumulation and *J*–*V* hysteresis of PSCs.

In summary, surface modifications of SnO_2_ ETLs have obtained prominent effects to the stability enhancement of PSCs against different environmental stresses, based on which we propose future research directions that were not fully accounted for in prior works, as detailed below.

### 4.1 Multifunctional Molecular Passivation

The defective buried interfaces of SnO_2_/perovskite are complex physical and chemical systems, which not only involve surface defects such as hydroxyls and dangling bonds but also the causal lattice mismatch, interfacial delamination, and etc. Synergistic solution to all these problems demands customized passivation reagents (e.g., molecules with multiple functional groups, field‐effect dipole molecules, polymers with special structural designs) with multiple functionality to simultaneously suppress surface defects of SnO_2_ ETLs while gaining integrity of interfacial mechanics, so as to improve stability and PV performance at the same time.

### 4.2 SnO_2_/Perovskite Integral Modifications

Even though SnO_2_ has been optimized with tailored modifications that give rise to stable performance of PSCs, it is after all inorganic in chemical composition and has large bulk modulus. It thus constitutes phenomenal lattice mismatch and large thermal expansivity discrepancy with organic‐contained perovskite films on top, thereby forming unavoidable interfacial residual stress upon film annealing. Therefore, constructing tailored interlayer designs is necessary to the strain relaxation at the buried interface, which is especially meaningful to the settings of flexible PSCs due to the massive thermally induced substrate deformation. Among the many manipulative routes, preburying volatile additives in SnO_2_ ETL can lead to effective regulation of electron mobility, interfacial stress, and defect density of perovskite film as a whole, thus forming gradient distribution of additive ingredients across the SnO_2_/perovskite interface.

### 4.3 Surface Composite‐Structure Designs

In terms of double‐ETL designs, while the bottom layer that interfaced with TCO substrates remain as planar structure, the top layer that contacts with perovskite can be selectively modified into porous or mesoporous morphology, which can accordingly enlarge the contact area with perovskite for enhanced interfacial adhesion while fulfilling the cascaded energy level alignments for efficient carrier extraction/transport.

## Conflict of Interest

The authors declare no conflict of interest.

## References

[1] H. Min , D. Y. Lee , J. Kim , G. Kim , K. S. Lee , J. Kim , M. J. Paik , Y. K. Kim , K. S. Kim , M. G. Kim , T. J. Shin , S. I. Seok , Nature 2021, 598, 444. 34671136 10.1038/s41586-021-03964-8

[2] D. Gao , B. Li , Z. Li , X. Wu , S. Zhang , D. Zhao , X. Jiang , C. Zhang , Y. Wang , Z. Li , N. Li , S. Xiao , W. C. H. Choy , A. K. Jen , S. Yang , Z. Zhu , Adv Mater 2022, 35, 2206387.

[3] N. Li , S. Tao , Y. Chen , X. Niu , C. K. Onwudinanti , C. Hu , Z. Qiu , Z. Xu , G. Zheng , L. Wang , Y. Zhang , L. Li , H. Liu , Y. Lun , J. Hong , X. Wang , Y. Liu , H. Xie , Y. Gao , Y. Bai , S. Yang , G. Brocks , Q. Chen , H. Zhou , Nat. Energy 2019, 4, 408.

[4] a) Z. Liu , L. Qiu , L. K. Ono , S. He , Z. Hu , M. Jiang , G. Tong , Z. Wu , Y. Jiang , D.-Y. Son , Y. Dang , S. Kazaoui , Y. Qi , Nat. Energy 2020, 5, 596;

[5] a) Q. Jiang , Y. Zhao , X. Zhang , X. Yang , Y. Chen , Z. Chu , Q. Ye , X. Li , Z. Yin , J. You , Nat. Photonics 2019, 13, 460;

[6] a) J. Xia , Y. Zhang , C. Xiao , K. G. Brooks , M. Chen , J. Luo , H. Yang , N. I. D. Klipfel , J. Zou , Y. Shi , X. Yao , J. Chen , J. M. Luther , H. Lin , A. M. Asiri , C. Jia , M. K. Nazeeruddin , Joule 2022, 6, 1689;

[7] a) B. Roose , C. M. Johansen , K. Dupraz , T. Jaouen , P. Aebi , U. Steiner , A. Abate , J. Mater. Chem. A 2018, 6, 1850;

[8] a) J. Liu , S. Li , S. Liu , Y. Chu , T. Ye , C. Qiu , Z. Qiu , X. Wang , Y. Wang , Y. Su , Y. Hu , Y. Rong , A. Mei , H. Han , Angew. Chem., Int. Ed. 2022, 61, e202202012; 10.1002/anie.202202012 35393733

[9] J. Song , H. Liu , W. Pu , Y. Lu , Z. Si , Z. Zhang , Y. Ge , N. Li , H. Zhou , W. Xiao , L. Wang , M. Sui , Energy Environ. Sci. 2022, 15, 4836.

[10] a) C. Altinkaya , E. Aydin , E. Ugur , F. H. Isikgor , A. S. Subbiah , M. De Bastiani , J. Liu , A. Babayigit , T. G. Allen , F. Laquai , A. Yildiz , S. De Wolf , Adv. Mater. 2021, 33, 2005504; 10.1002/adma.202005504 33660306

[11] V. B. Kamble , A. M. Umarji , AIP Adv. 2013, 3, 082120.

[12] X. Chen , L. Liu , F. Huang , Chem. Soc. Rev. 2015, 44, 1861. 25590565 10.1039/c4cs00330f

[13] J. Tauc , R. Grigorovici , A. Vancu , Phys. Status Solidi B 1966, 15, 627.

[14] a) W. Ke , G. Fang , Q. Liu , L. Xiong , P. Qin , H. Tao , J. Wang , H. Lei , B. Li , J. Wan , G. Yang , Y. Yan , J. Am. Chem. Soc. 2015, 137, 6730; 25987132 10.1021/jacs.5b01994

[15] D. Wang , S.-C. Chen , Q. Zheng , J. Mater. Chem. C 2019, 7, 12204.

[16] L. Xiong , M. Qin , G. Yang , Y. Guo , H. Lei , Q. Liu , W. Ke , H. Tao , P. Qin , S. Li , H. Yu , G. Fang , J. Mater. Chem. A 2016, 4, 8374.

[17] R. Yuan , B. Cai , Y. Lv , X. Gao , J. Gu , Z. Fan , X. Liu , C. Yang , M. Liu , W.-H. Zhang , Energy Environ. Sci. 2021, 14, 5074.

[18] Y. Wang , C. Duan , J. Li , W. Han , M. Zhao , L. Yao , Y. Wang , C. Yan , T. Jiu , ACS Appl. Mater. Interfaces 2018, 10, 20128. 29785850 10.1021/acsami.8b03444

[19] J. J. Yoo , G. Seo , M. R. Chua , T. G. Park , Y. Lu , F. Rotermund , Y. K. Kim , C. S. Moon , N. J. Jeon , J. P. Correa-Baena , V. Bulovic , S. S. Shin , M. G. Bawendi , J. Seo , Nature 2021, 590, 587. 33627807 10.1038/s41586-021-03285-w

[20] a) Z. Song , W. Bi , X. Zhuang , Y. Wu , B. Zhang , X. Chen , C. Chen , Q. Dai , H. Song , Sol. RRL 2020, 4, 1900266;

[21] L. Kavan , L. Steier , M. Grätzel , J. Phys. Chem. C 2016, 121, 342.

[22] R. M. Pasquarelli , D. S. Ginley , R. O’Hayre , Chem. Soc. Rev. 2011, 40, 5406. 21687838 10.1039/c1cs15065k

[23] a) C. Xu , Z. Liu , Q. Sun , E.-C. Lee , Sol. Energy 2021, 214, 280;

[24] a) D. P. McMeekin , P. Holzhey , S. O. Furer , S. P. Harvey , L. T. Schelhas , J. M. Ball , S. Mahesh , S. Seo , N. Hawkins , J. Lu , M. B. Johnston , J. J. Berry , U. Bach , H. J. Snaith , Nat. Mater. 2022, 22, 73; 36456873 10.1038/s41563-022-01399-8

[25] T. Bu , L. K. Ono , J. Li , J. Su , G. Tong , W. Zhang , Y. Liu , J. Zhang , J. Chang , S. Kazaoui , F. Huang , Y.-B. Cheng , Y. Qi , Nat. Energy 2022, 7, 528.

[26] L. Qiu , Z. Liu , L. K. Ono , Y. Jiang , D. Y. Son , Z. Hawash , S. He , Y. Qi , Adv. Funct. Mater. 2018, 29, 1806779.

[27] L. Xiong , J. Li , F. Ye , H. Wang , Y. Guo , X. Ming , Q. Chen , S. Zhang , R. Xie , Z. Chen , Y. Lv , G. Hu , Y. He , G. Fang , Adv. Funct. Mater. 2021, 31, 2103949.

[28] Y. Rong , Y. Hu , A. Mei , H. Tan , M. I. Saidaminov , S. I. Seok , M. D. McGehee , E. H. Sargent , H. Han , Science 2018, 361, eaat8235. 30237326 10.1126/science.aat8235

[29] a) B. Roose , J.-P. C. Baena , K. C. Gödel , M. Graetzel , A. Hagfeldt , U. Steiner , A. Abate , Nano Energy 2016, 30, 517;

[30] P. F. Méndez , S. K. M. Muhammed , E. M. Barea , S. Masi , I. Mora-Seró , Sol. RRL 2019, 3, 1900191.

[31] Q. Dong , J. Li , Y. Shi , M. Chen , L. K. Ono , K. Zhou , C. Zhang , Y. Qi , Y. Zhou , N. P. Padture , Adv. Mater. 2019, 9, 1900834.

[32] S. You , H. Zeng , Z. Ku , X. Wang , Z. Wang , Y. Rong , Y. Zhao , X. Zheng , L. Luo , L. Li , S. Zhang , M. Li , X. Gao , X. Li , Adv. Mater. 2020, 32, 2003990. 10.1002/adma.202003990 32954577

[33] H. Dong , J. Wang , X. Li , W. Liu , T. Xia , D. Yao , L. Zhang , C. Zuo , L. Ding , F. Long , ACS Appl. Mater. Interfaces 2022, 14, 34143. 10.1021/acsami.2c08662 35820159

[34] a) X. Zhang , Y. Rui , Y. Wang , J. Xu , H. Wang , Q. Zhang , P. Müller-Buschbaum , J. Power Sources 2018, 402, 460;

[35] X. Zhang , Y. Rui , J. Yang , L. Wang , Y. Wang , J. Xu , Appl. Surf. Sci. 2019, 463, 679.

[36] H. Huang , X. Liu , M. Duan , J. Ji , H. Jiang , B. Liu , S. Sajid , P. Cui , D. Wei , Y. Li , M. Li , ACS Appl. Energy Mater. 2020, 3, 5039.

[37] R. Keshtmand , M. R. Zamani-Meymian , F. Mohamadkhani , N. Taghavinia , Sol. Energy 2021, 228, 253.

[38] H. Bi , B. Liu , D. He , L. Bai , W. Wang , Z. Zang , J. Chen , Chem. Eng. J. 2021, 418, 129375.

[39] S. S. Mali , J. V. Patil , H. Arandiyan , C. K. Hong , J. Mater. Chem. A 2019, 7, 17516.

[40] H. Feng , S. Liu , G. Tang , L. Zhang , W. Xie , J. Mater. Chem. C 2021, 9, 13748.

[41] K. Jung , D. H. Kim , J. Kim , S. Ko , J. W. Choi , K. C. Kim , S.-G. Lee , M.-J. Lee , J. Mater. Sci. Technol. 2020, 59, 195.

[42] Z. Xiong , X. Chen , B. Zhang , G. O. Odunmbaku , Z. Ou , B. Guo , K. Yang , Z. Kan , S. Lu , S. Chen , N. A. N. Ouedraogo , Y. Cho , C. Yang , J. Chen , K. Sun , Adv. Mater. 2022, 34, 2106118. 10.1002/adma.202106118 34862820

[43] Y. Dong , W. Shen , W. Dong , C. Bai , J. Zhao , Y. Zhou , F. Huang , Y. B. Cheng , J. Zhong , Adv. Mater. 2022, 12, 2200417.

[44] Z. Zheng , F. Li , J. Gong , Y. Ma , J. Gu , X. Liu , S. Chen , M. Liu , Adv. Mater. 2022, 34, 2109879. 10.1002/adma.202109879 35384082

[45] H. Liu , Z. Zhang , Z. Su , W. Zuo , Y. Tang , F. Yang , X. Zhang , C. Qin , J. Yang , Z. Li , M. Li , Adv. Sci. 2022, 9, 2105739. 10.1002/advs.202105739 PMC9008411 35212188

[46] L. Yang , J. Feng , Z. Liu , Y. Duan , S. Zhan , S. Yang , K. He , Y. Li , Y. Zhou , N. Yuan , J. Ding , S. F. Liu , Adv. Mater. 2022, 34, 2201681. 10.1002/adma.202201681 35435279

[47] a) A. Ulman , Chem. Rev. 1996, 96, 1533; 11848802 10.1021/cr9502357

[48] F. Ali , C. Roldán-Carmona , M. Sohail , M. K. Nazeeruddin , Adv. Energy Mater. 2020, 10, 2002989.

[49] Z. Dai , S. K. Yadavalli , M. Chen , A. Abbaspourtamijani , Y. Qi , N. P. Padture , Science 2021, 372, 618. 33958474 10.1126/science.abf5602

[50] Z. Dai , S. Li , X. Liu , M. Chen , C. E. Athanasiou , B. W. Sheldon , H. Gao , P. Guo , N. P. Padture , Adv. Mater. 2022, 34, 2205301. 10.1002/adma.202205301 36148590

[51] a) H. Wang , C. Zhu , L. Liu , S. Ma , P. Liu , J. Wu , C. Shi , Q. Du , Y. Hao , S. Xiang , H. Chen , P. Chen , Y. Bai , H. Zhou , Y. Li , Q. Chen , Adv. Mater. 2019, 31, 1904408; 10.1002/adma.201904408 31617644

[52] Q. Zhou , D. He , Q. Zhuang , B. Liu , R. Li , H. Li , Z. Zhang , H. Yang , P. Zhao , Y. He , Z. Zang , J. Chen , Adv. Funct. Mater. 2022, 32, 2205507.

[53] J. Wu , Y. Cui , B. Yu , K. Liu , Y. Li , H. Li , J. Shi , H. Wu , Y. Luo , D. Li , Q. Meng , Adv. Funct. Mater. 2019, 29, 1905336.

[54] Z. Ni , H. Jiao , C. Fei , H. Gu , S. Xu , Z. Yu , G. Yang , Y. Deng , Q. Jiang , Y. Liu , Y. Yan , J. Huang , Nat. Energy 2021, 7, 65.

[55] P. Wang , R. Li , B. Chen , F. Hou , J. Zhang , Y. Zhao , X. Zhang , Adv. Mater. 2020, 32, 1905766. 10.1002/adma.201905766 31899829

[56] a) Y. Hou , X. Chen , S. Yang , C. Li , H. Zhao , H. G. Yang , Adv. Funct. Mater. 2017, 27, 1700878;

[57] S. Tan , T. Huang , I. Yavuz , R. Wang , T. W. Yoon , M. Xu , Q. Xing , K. Park , D. K. Lee , C. H. Chen , R. Zheng , T. Yoon , Y. Zhao , H. C. Wang , D. Meng , J. Xue , Y. J. Song , X. Pan , N. G. Park , J. W. Lee , Y. Yang , Nature 2022, 605, 268. 35292753 10.1038/s41586-022-04604-5

[58] J. Ye , Y. Li , A. A. Medjahed , S. Pouget , D. Aldakov , Y. Liu , P. Reiss , Small 2021, 17, 2005671. 10.1002/smll.202005671 33369877

[59] S. Song , G. Kang , L. Pyeon , C. Lim , G.-Y. Lee , T. Park , J. Choi , ACS Energy Lett. 2017, 2, 2667.

[60] J. Liu , Y. Guo , M. Zhu , Y. Li , X. Li , J. Power Sources 2020, 476, 228648.

[61] M. M. Lee , J. Teuscher , T. Miyasaka , T. N. Murakami , H. J. Snaith , Science 2012, 338, 643. 23042296 10.1126/science.1228604

[62] Q. Dong , C. Zhu , M. Chen , C. Jiang , J. Guo , Y. Feng , Z. Dai , S. K. Yadavalli , M. Hu , X. Cao , Y. Li , Y. Huang , Z. Liu , Y. Shi , L. Wang , N. P. Padture , Y. Zhou , Nat. Commun. 2021, 12, 973. 33579915 10.1038/s41467-021-21292-3 PMC7881119

[63] a) C. Wang , L. Guan , D. Zhao , Y. Yu , C. R. Grice , Z. Song , R. A. Awni , J. Chen , J. Wang , X. Zhao , Y. Yan , ACS Energy Lett. 2017, 2, 2118;

[64] T. Bu , J. Li , F. Zheng , W. Chen , X. Wen , Z. Ku , Y. Peng , J. Zhong , Y. B. Cheng , F. Huang , Nat. Commun. 2018, 9, 4609. 30389948 10.1038/s41467-018-07099-9 PMC6214926

[65] a) P. Schulz , J. O. Tiepelt , J. A. Christians , I. Levine , E. Edri , E. M. Sanehira , G. Hodes , D. Cahen , A. Kahn , ACS Appl. Mater. Interfaces 2016, 8, 31491; 27933974 10.1021/acsami.6b10898

[66] J. Kim , G. Kim , T. K. Kim , S. Kwon , H. Back , J. Lee , S. H. Lee , H. Kang , K. Lee , J. Mater. Chem. A 2014, 2, 17291.

[67] a) B. Roose , Q. Wang , A. Abate , Adv. Energy Mater. 2019, 9, 1803140;

[68] a) W. Chen , F. Z. Liu , X. Y. Feng , A. B. Djurišić , W. K. Chan , Z. B. He , Adv. Energy Mater. 2017, 7, 1700722;

[69] a) X. Ye , H. Ling , R. Zhang , Z. Wen , S. Hu , T. Akasaka , J. Xia , X. Lu , J. Power Sources 2020, 448, 227419;

[70] a) X. Wang , L.-L. Deng , L.-Y. Wang , S.-M. Dai , Z. Xing , X.-X. Zhan , X.-Z. Lu , S.-Y. Xie , R.-B. Huang , L.-S. Zheng , J. Mater. Chem. A 2017, 5, 1706;

[71] a) Q. Luo , H. Chen , Y. Lin , H. Du , Q. Hou , F. Hao , N. Wang , Z. Guo , J. Huang , Adv. Funct. Mater. 2017, 27, 1702090;

[72] a) J. J. M. Vequizo , M. Yokoyama , M. Ichimura , A. Yamakata , Appl. Phys. Express 2016, 9, 067101;

[73] J. Song , E. Zheng , J. Bian , X.-F. Wang , W. Tian , Y. Sanehira , T. Miyasaka , J. Mater. Chem. A 2015, 3, 10837.

[74] W.-Q. Wu , D. Chen , Y.-B. Cheng , R. A. Caruso , Sol. RRL 2017, 1, 1700117.

[75] X. Liu , Y. Zhang , L. Shi , Z. Liu , J. Huang , J. S. Yun , Y. Zeng , A. Pu , K. Sun , Z. Hameiri , J. A. Stride , J. Seidel , M. A. Green , X. Hao , Adv. Mater. 2018, 8, 1800138.

[76] S. Akin , ACS Appl. Mater. Interfaces 2019, 11, 39998. 31596065 10.1021/acsami.9b13876

[77] P. Hang , J. Xie , G. Li , Y. Wang , D. Fang , Y. Yao , D. Xie , C. Cui , K. Yan , J. Xu , D. Yang , X. Yu , iScience 2019, 21, 217. 31675551 10.1016/j.isci.2019.10.021 PMC6838471

[78] X. Chen , W. Xu , Z. Shi , G. Pan , J. Zhu , J. Hu , X. Li , C. Shan , H. Song , Nano Energy 2021, 80, 105564.

[79] M. Abuhelaiqa , N. Shibayama , X.-X. Gao , H. Kanda , M. K. Nazeeruddin , ACS Appl. Energy Mater. 2021, 4, 3424.

[80] Z. Qin , Y. Chen , X. Wang , N. Wei , X. Liu , H. Chen , Y. Miao , Y. Zhao , Adv. Mater. 2022, 34, 2203143. 10.1002/adma.202203143 35732580

[81] G. Mathiazhagan , A. Seeber , T. Gengenbach , S. Mastroianni , D. Vak , A. S. R. Chesman , M. Gao , D. Angmo , A. Hinsch , Sol. RRL 2020, 4, 2000262.

[82] J. Chen , X. Zhao , S. G. Kim , N. G. Park , Adv. Mater. 2019, 31, 1902902. 10.1002/adma.201902902 31402565

[83] J. Dagar , S. Castro-Hermosa , G. Lucarelli , F. Cacialli , T. M. Brown , Nano Energy 2018, 49, 290.

[84] C. Tian , K. Lin , J. Lu , W. Feng , P. Song , L. Xie , Z. Wei , Small Methods 2019, 4, 1900476.

